# A KIF1C-CNBP motor-adaptor complex for trafficking mRNAs to cell protrusions

**DOI:** 10.1016/j.celrep.2025.115346

**Published:** 2025-02-20

**Authors:** Konstadinos Moissoglu, Tianhong Wang, Alexander N. Gasparski, Michael Stueland, Elliott L. Paine, Lisa M. Jenkins, Stavroula Mili

**Affiliations:** 1 Laboratory of Cellular and Molecular Biology, Center for Cancer Research, National Cancer Institute, NIH, Bethesda, MD 20892, USA; 2 Laboratory of Cell Biology, Center for Cancer Research, National Cancer Institute, NIH, Bethesda, MD 20892, USA; 3 Lead contact

## Abstract

mRNA localization to subcellular compartments is a widely used mechanism that functionally contributes to numerous processes. mRNA targeting can be achieved upon recognition of RNA cargo by molecular motors. However, our molecular understanding of how this is accomplished is limited, especially in higher organisms. We focus on a pathway that targets mRNAs to peripheral protrusions of mammalian cells and which is important for cell migration. Trafficking occurs through active transport on microtubules, mediated by the KIF1C kinesin. Here, we identify the RNA-binding protein CNBP as a factor required for mRNA localization to protrusions. CNBP binds directly to GA-rich sequences in the 3′ UTR of protrusion-targeted mRNAs. CNBP also interacts with KIF1C and is required for KIF1C recruitment to mRNAs and their trafficking on microtubules to the periphery. This work provides a molecular mechanism for KIF1C recruitment to mRNA cargo and reveals a motor-adaptor complex for mRNA transport to cell protrusions.

## In brief

Moissoglu et al. study the mechanisms of mRNA targeting to mammalian cell protrusions. They show that the RNA-binding protein CNBP binds 3′ UTR GA-rich sequences in protrusion-localized mRNAs and recruits the KIF1C kinesin for mRNA trafficking on microtubules. This work reveals a new motor-adaptor complex for microtubule-based mRNA transport.

## Graphical Abstract

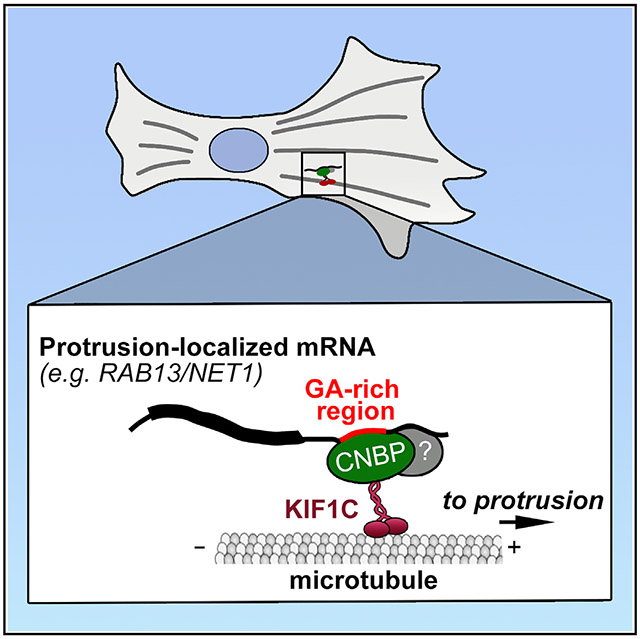

## INTRODUCTION

Localization of mRNAs to specific subcellular compartments is a widely used mechanism that critically impacts the spatiotemporal regulation of gene expression and affects various functional outcomes.^1-3^ Biological processes that rely on the localization of mRNAs include cell fate determination, embryonic patterning, neuronal outgrowth, synaptic plasticity, and cell migration.^3-6^ Accordingly, mRNA targeting and local regulation are important for ensuring proper organismal development and are deregulated in a variety of neurodegenerative and neuromuscular disorders.^7-9^

A variety of mechanisms have been described that can lead to asymmetric mRNA distributions. These include random diffusion coupled with local entrapment, transcript degradation coupled to localized protection, and active transport to specific destinations.^9-11^ The latter often occurs along cytoskeletal elements and relies on the action of molecular motors, such as kinesins, dynein, and myosin.^12^ Individual mRNA molecules can serve as cargo of molecular motors, or they can co-assemble into RNA granules for the coordinated transport of multiple mRNAs.^13,14^ Alternatively, hitchhiking of RNAs onto endosomes or lysosomes can support long-range RNA movements.^15-17^

These various trafficking mechanisms generally involve *cis*-acting RNA elements that are either necessary or sufficient to confer a specific distribution pattern and are usually found in the 3′ UTRs of localized transcripts. Such localization elements have been sporadically identified for individual transcripts.^18^ Recent studies geared to identify such sequences in a high-throughput manner have additionally revealed shorter or longer regulatory elements involved in mRNA targeting.^19-21^ How these RNA elements mediate the interaction of an mRNA with the machinery that mediates local accumulation is not well understood, and detailed molecular understanding is available for only a few cases.

For example, in yeast, localization elements within the *Ash1* mRNA are recognized by the She2p RNA-binding protein (RBP), which recruits the type V myosin motor, Myo4p, through the She3p adaptor.^22^ In another case, the *Drosophila*-specific RBP Egalitarian (Egl) links various localized RNA cargoes to the dynein motor. Egl binds to RNA stem loops that mediate polarized transport and, additionally, associates with the dynein adaptor BICD2 and the dynein light-chain subunit of the dynein motor complex.^23^ These components are sufficient to support directed RNA transport in *in vitro* reconstitution experiments, thus defining a minimal transport-competent complex for directed transport to the minus ends of microtubules.^24,25^ A variety of other RNA cargoes rely on kinesin motors for trafficking toward the plus ends of microtubules. In the case of the *Drosophila* oskar mRNA, the atypical, RNA-binding tropomyosin (Tm1-I/C; aTm1) serves to stabilize the interaction of kinesin heavy chain (KHC) with oskar mRNA^26,27^ and regulates kinesin activity to allow coordination with dynein-mediated transport at different stages of oocyte maturation.^28^ While a number of other RBPs have been implicated in transport events, the exact RNA signals recognized and/or the links to molecular motors have been less well defined, especially in mammalian systems.^29-33^

In mammalian mesenchymal cells, a robust localization pathway targets mRNAs to peripheral protrusive regions.^34,35^ mRNAs targeted to cell protrusions encode regulators of cell migration, and their local translation in peripheral regions ensures efficient cell movement and invasion. Specifically, local protein synthesis promotes co-translational interactions of the nascent proteins that favor promigratory phenotypes.^36,37^ As described in other cases, the regulatory information directing protrusion mRNA targeting is found within the 3′ UTRs, which are sufficient to direct peripheral localization of otherwise diffuse mRNAs. Moreover, specific GA-rich regions have critical roles, and interfering with or deleting such GA-rich regions is sufficient to disrupt peripheral localization and perturb cell movement in various systems.^36-39^ Localization to the periphery requires the microtubule cytoskeleton and, in particular, a subset of stable, detyrosinated microtubules.^35,40,41^ Active trafficking on microtubules is mediated though the kinesin-3 family member KIF1C. KIF1C co-traffics with individual mRNAs along linear paths and is required for their localization, suggesting that it is the main kinesin motor supporting this localization pathway.^42^ KIF1C can associate with the RBP muscleblind-like 1 (MBNL1) and affects RNA trafficking to neurites.^43^ Nevertheless, how KIF1C recognizes and is recruited to protrusion-localized RNA cargoes has been unclear.

Here, we identify the RBP CNBP as the factor recognizing GA-rich regions of protrusion-targeted mRNAs. We demonstrate that CNBP participates in this localization pathway by interacting with KIF1C and serving as an adaptor that recruits the motor to RNA cargo. Our data present a novel motor-adaptor complex that supports the trafficking of mRNAs to cellular protrusions.

## RESULTS

### Identification of proteins binding to localization sequences of protrusion-localized mRNAs

**Figure 1.**
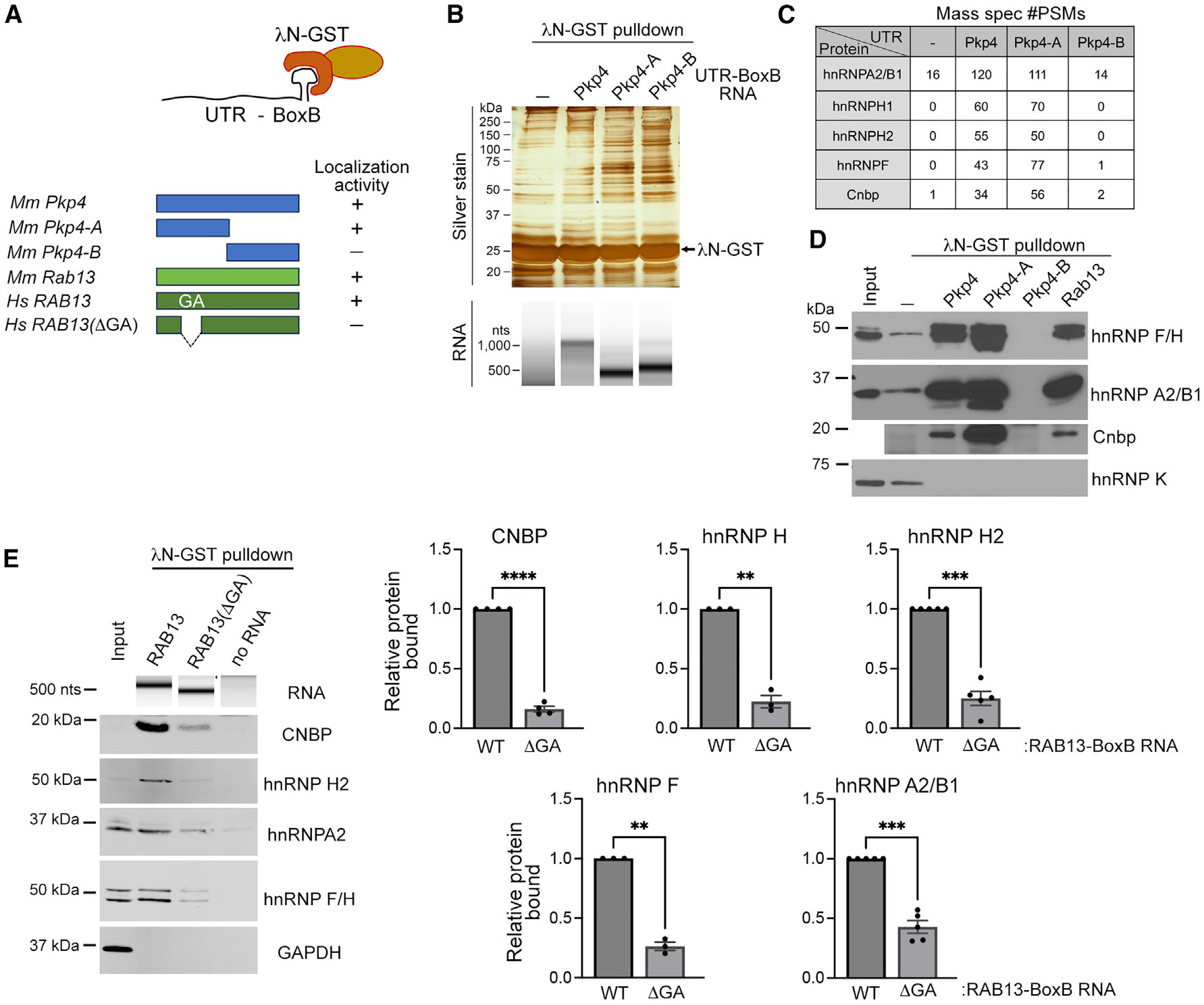
Identification of proteins binding to localization regions of protrusion-localized mRNAs (A) Schematic of BoxB-containing UTR fragments used in λN-GST pull-down assays. Localization activity is indicated based on prior reports.^35,36^ (B) Pull-down with the indicated UTRs from mouse NIH/3T3 fibroblast lysates. Recovered proteins and RNAs were analyzed through silver staining and TapeStation, respectively. (C) Mass spectrometry (mass spec) identification of RBPs preferentially bound to localization-competent Pkp4 UTR fragments. (D) Western blot analysis of the indicated proteins in RNA pull-down samples from NIH/3T3 fibroblast lysates with mouse Pkp4 or Rab13 UTRs. (E) RNA pull-down samples, from MDA-MB-231 cell lysates, with human RAB13 UTRs were analyzed on TapeStation to detect recovered RNAs or by western blot to detect the indicated proteins. Graphs indicate the quantifications of bound protein to the wild-type (WT) or truncated (ΔGA) UTRs. *n* = 3–5. Error bars: SEM. **p < 0.01, ***p < 0.001, and ****p < 0.0001 by paired t test. See also Table S1.

Several protrusion-localized mRNAs are targeted to peripheral subcellular locations by associating with the KIF1C kinesin.^42^ Sequences within the 3′ UTRs are necessary and sufficient for peripheral targeting through this mechanism.^36,37,39^ We sought to identify factors that connect these mRNAs to the KIF1C motor. For this, we carried out an unbiased mass spectrometry identification of proteins that bind to the 3′ UTR of such mRNAs. To narrow down to proteins that might be relevant to the localization pathway, we identified proteins that bind to the full-length mouse *Pkp4* 3′ UTR or to 3′ UTR fragments that have been shown in prior studies to retain, or not, localization activity^35^ (Figure 1A; Pkp4-A and Pkp4-B, respectively). These RNA fragments were generated through *in vitro* transcription and designed to additionally contain, at their 3′ end, a BoxB hairpin sequence from the λ bacteriophage. The BoxB sequence is recognized by a peptide of the λN protein, which, when fused to glutathione S-transferase (GST), allows immobilization of BoxB-containing RNAs on glutathione beads (Figure 1A). Incubation with cellular lysates can then allow the pull-down of proteins that bind to specific RNAs.

Multiple proteins that specifically bound to *Pkp4* RNA fragments could be visualized by silver staining, despite a high degree of background binding even in the absence of any immobilized RNA (Figure 1B). These were further identified by mass spectrometry (Figure 1C; Table S1). We focused on proteins that were preferentially associated with the localization-competent UTR sequences (Pkp4 and Pkp4-A) over either background binding or binding to sequences that do not support localization (Pkp4-B) (Figure 1C; Table S1). Several RBPs were identified from mouse NIH/3T3 cell lysates that exhibited these characteristics, including hnRNPA2/B1, hnRNPH1, hnRNPH2, hnRNPF, and CNBP. To validate the identification of these RBPs, similar pull-downs were performed, and the recovered proteins were analyzed by immunoblotting. Indeed, all the candidate proteins were specifically enriched in the pull-downs of localization-competent Pkp4 RNA fragments (Figure 1D). Other RBPs, such as hnRNPK, were not detected in pull-downs with any Pkp4 RNA sequences, underscoring the specificity of the identified interactions.

Multiple mRNAs are targeted to protrusions through the same KIF1C-dependent mechanism. This localization pathway is also conserved across species.^36,39^ We thus extended our analysis to assess whether the identified RBPs also associated with the 3′ UTR of another protrusion-localized mRNA, the mouse *Rab13* mRNA. To further explore conservation among species, we performed pull-down assays using the 3′ UTR of the human *RAB13* mRNA and cell lysates from the human MDA-MB-231 cell line. Indeed, we found that the same complement of RBPs associated with the mouse *Rab13* 3′ UTR (Figure 1D) and that the same interactions were conserved in a human system (Figure 1E; human *RAB13* 3′ UTR).

In several cases, localization to protrusions relies on GA-rich regions within the 3′ UTRs of targeted mRNAs.^36,37,39,44^ In the case of the human *RAB13* mRNA, when a specific ~50 nt GA-rich region within the 3′ UTR is deleted, or when its function is interfered with using antisense oligonucleotides (oligos), peripheral *RAB13* mRNA localization is impaired.^36^ Therefore, to further explore the potential involvement of the identified RBPs to the localization mechanism, we tested whether the *RAB13* GA-rich sequence is important for their binding. For this, we generated a truncated human *RAB13* 3′ UTR fragment missing the GA-rich sequence and performed pull-down assays using human cell lysates. Interestingly, all identified RBPs exhibited significantly reduced binding to the truncated *RAB13* 3′ UTR compared to the full-length, wild-type (WT) counterpart (Figure 1E). Taken together, these results indicate that RNA sequences that are necessary and sufficient for mRNA protrusion localization specifically associate with a group of RBPs, making these RBPs candidate participants in the localization mechanism.

### CNBP is required for localization of mRNAs to cytoplasmic protrusions

**Figure 2.**
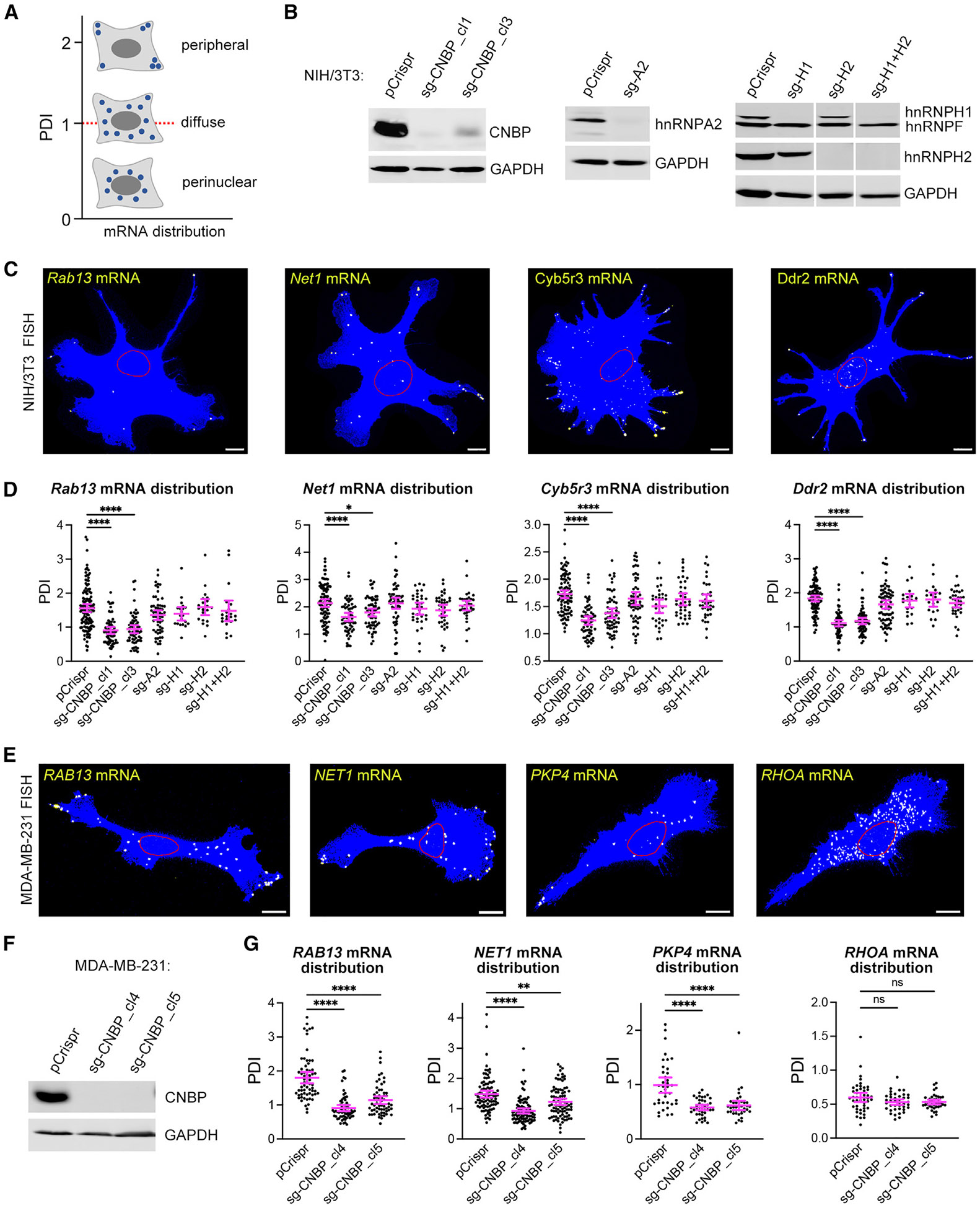
CNBP is required for localization of mRNAs to cytoplasmic protrusions (A) Schematic depicting quantification of RNA distributions through PDI metric. Higher PDI values indicate more peripheral RNA distribution in a cell. (B) Western blot of NIH/3T3 cell lines CRISPR edited with the indicated sgRNAs. (C) Representative FISH images of control (pCrispr) NIH/3T3 cells detecting the indicated mRNAs. Blue: cell mask; red line: outline of nucleus (based on DAPI stain); yellow: RNA. Scale bar: 10 μm. (D) PDI quantifications of *Rab13*, *Net1*, *Cyb5r3*, and *Ddr2* mRNA distributions from the indicated CRISPR-edited NIH/3T3 cell lines. Only CNBP loss leads to less peripheral RNA distributions. *n* = 20–118 cells. Error bars: SEM. *p < 0.05 and ****p < 0.0001 by Kruskal-Wallis test with Dunn’s multiple comparisons test. (E) Representative FISH images of control (pCrispr) MDA-MB-231 cells detecting the indicated mRNAs. Blue: cell mask; red line: outline of nucleus (based on DAPI stain); yellow: RNA. Scale bar: 10 μm. (F) Western blot of CNBP levels in CRISPR-edited clonal cell lines. (G) PDI quantifications of *RAB13*, *NET1*, and *PKP4* and *RHOA* mRNA distributions from the indicated CRISPR-edited MDA-MB-231 cell lines. *n* = 38–64 cells. Error bars: SEM. **p < 0.01, ****p < 0.0001, and ns, non-significant, by Kruskal-Wallis test with Dunn’s multiple comparisons test. See also Figures S1 and S2.

To evaluate the role of these RBPs in mRNA localization at protrusions, we knocked down their expression in mouse NIH/3T3 cells using small interfering RNAs (siRNAs). Transient knockdown led to a significant decrease of the corresponding RBPs (Figure S1A). To assess whether this affected mRNA localization to protrusions, we visualized the distribution of protrusion-localized mRNAs (*Rab13* and *Net1*) with fluorescence *in situ* hybridization (FISH) and quantified it using a previously described peripheral distribution index (PDI)^45^ (Figures 2A and S1B). Briefly, higher PDI values indicate a more peripheral distribution, while lower values denote a perinuclear bias^45^ (Figure 2A; see also the STAR Methods). Interestingly, only the knockdown of CNBP significantly reduced the peripheral targeting of both *Rab13* and *Net1* mRNAs, as indicated by a reduced PDI index, while the knockdown of hnRNPA2, hnRNPH1, or hnRNPH2 (individually or in combination) did not significantly or consistently affect peripheral mRNA targeting (Figure S1B).

To independently confirm these observations and rule out the possibility that residual amounts of hnRNP proteins might prevent us from observing any additional functional contributions, we ablated the expression of these RBPs using CRISPR-Cas9 genome editing. single guide RNAs (sgRNAs) targeting each candidate RBP were used, and immunoblot analysis verified that this led to undetectable protein levels of the corresponding RBPs (Figure 2B). We again assessed the distribution of protrusion-localized mRNAs and extended our analysis to include additional transcripts (*Rab13*, *Net1*, *Cyb5r3*, and *Ddr2*) (Figure 2C). Consistent with the results obtained by transient knockdown, the loss of hnRNPA2, hnRNPH1, or hnRNPH2 did not affect the distribution of any of the tested mRNAs (Figure 2D). CNBP loss, on the other hand, significantly reduced the peripheral localization of all tested mRNAs, and the effect was consistently observed in two independently isolated clones (Figure 2D).

To further address whether this is a conserved CNBP role across species, we used CRIPSR to knock out CNBP expression in the human MDA-MB-231 cell line. Two clonal cell populations were isolated, exhibiting undetectable CNBP expression (Figure 2F), and the distribution of several mRNAs was assessed (Figures 2E-2G, S1C, and S1D). Again, CNBP loss significantly reduced the peripheral targeting of all protrusion-targeted mRNAs tested (*RAB13*, *NET1*, *PKP4*, and *TRAK2*) while not affecting the distribution of other mRNAs (*RHOA* and *RPS20*), which rely on distinct mechanisms and factors for their cytoplasmic distribution (Figures 2E-2G, S1C, and S1D). Additionally, the loss of CNBP did not alter the overall *RAB13* and *NET1* mRNA levels or the corresponding protein amounts (Figure S2), indicating that at least for these transcripts, CNBP plays a specific role in their localization mechanism. Overall, we conclude that in both mouse and human cells, CNBP is important for the localization of mRNAs to cytoplasmic protrusions.

### CNBP binds directly to protrusion localized mRNAs through localization sequences in the 3′ UTR

**Figure 3.**
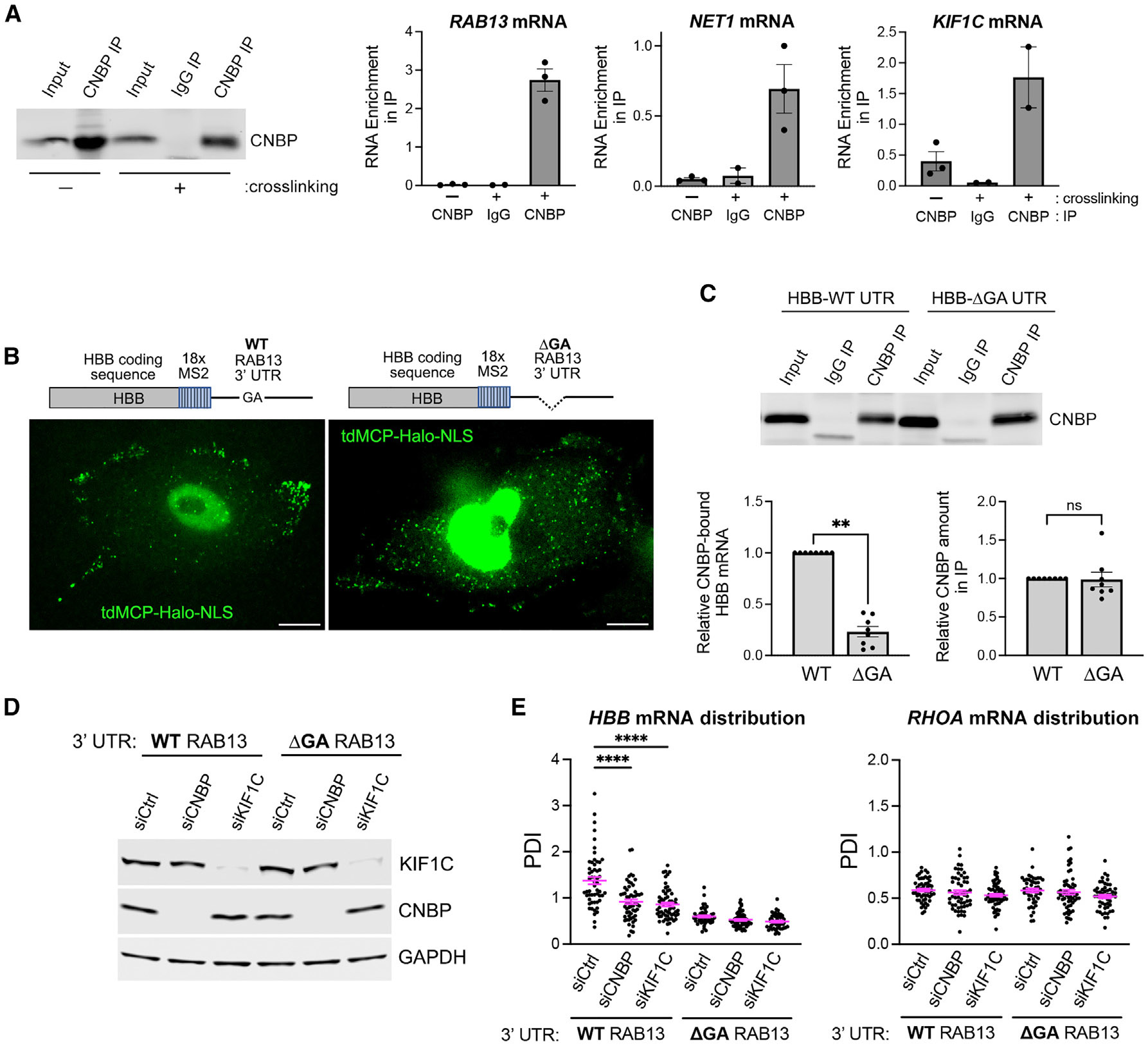
CNBP binds directly to protrusion-localized mRNAs through GA-rich regions (A) MDA-MB-231 cells were crosslinked, or not, with formaldehyde, CNBP was immunoprecipitated, and associated RNAs detected by ddPCR. Left: western blot to detect CNBP. Right graphs: amount of indicated mRNAs in immunoprecipitates. Values are expressed as enrichment relative to the corresponding mRNA amount in the input. *n* = 3 independent replicates. Error bars: SEM. (B) Schematics of MS2 reporter RNAs containing the μ-globin (HBB) coding sequence, 18 MS2 hairpins, and the WT or truncated RAB13 UTRs. The images are snapshots of cells stably expressing each reporter and tdMCP-Halo-NLS. Cells were labeled with Halo ligand and visualized live. The green spots in the cytoplasm correspond to individual mRNAs. The nuclear signal reflects excess tdMCP-Halo-NLS protein. Scale bar: 10 μm. (C) CNBP immunoprecipitation from cells expressing the indicated reporter RNAs. Top: western blot to detect CNBP. Bottom graphs: amount of reporter mRNA, or CNBP protein, in immunoprecipitates. *n* = 8 independent replicates. Error bars: SEM. **p < 0.01 and ns, non-significant, by Wilcoxon matched-pairs signed-rank test. (D) Western blot of CNBP and KIF1C levels in siRNA-treated cells expressing reporter RNAs with the indicated WT or ΔGA RAB13 UTRs. (E) PDI quantifications from the indicated siRNA-treated cells. Distributions are shown of reporter RNA (HBB) with WT or ΔGA RAB13 UTR, and of endogenous *RHOA* mRNA in the same cells. *n* = 48–62 cells. Error bars: SEM. ****p < 0.0001 by Kruskal-Wallis test with Dunn’s multiple comparisons test. See also Figure S3.

The association of CNBP with localization-competent, *in-vitro*-transcribed mRNA fragments suggested that CNBP might exert its role in RNA targeting by directly binding to localization sequences. To address this, we first explored whether we could detect an interaction of CNBP with protrusion-localized mRNAs *in vivo*. To avoid reassociations that can occur during cell lysis, we performed *in vivo* crosslinking with the short-range crosslinker formaldehyde to induce covalent bonds between RNAs and proteins that are in close contact with them. Then, under denaturing conditions, we immunoprecipitated CNBP and measured the amount of co-precipitated protrusion-localized mRNAs using digital droplet PCR (ddPCR). Indeed, several protrusion-localized mRNAs (*RAB13*, *NET1*, and *KIF1C*) were enriched in CNBP immunoprecipitates (Figure 3A). These associations were specific since no binding was observed when immunoprecipitations were carried out with control immunoglobulin (Ig)G. Additionally, in the absence of crosslinking, even though similar amounts of CNBP were recovered (Figure 3A, left), no co-precipitated mRNAs were detected (Figure 3A, right graphs), indicating that under these denaturing conditions, interactions were disrupted unless the binding partners were previously covalently linked with formaldehyde due to their close proximity in cells. Therefore, CNBP associates with protrusion-localized mRNAs *in vivo*, likely in a direct manner.

To address whether this interaction requires RNA sequences important for protrusion localization, we examined the ability of CNBP to bind to two reporter mRNAs. One contained the full-length 3′ UTR of human *RAB13* mRNA, while the other contained a truncated UTR (ΔGA) missing the ~50 nt GA-rich sequence that has been shown to be important for peripheral localization^36^ (Figure 3B). These reporters also contained the β-globin (HBB) coding sequence and 18 hairpins of the MS2 bacteriophage for *in vivo* visualization upon co-expression of the MS2 coat protein (MCP) fused to HaloTag and a nuclear localization signal (NLS) (tdMCP-Halo-NLS) (Figure 3B, schematics). The reporters were stably integrated under an inducible promoter, and their expression was induced for a few hours to achieve relatively low levels of expression. Following *in vivo* crosslinking with formaldehyde, CNBP was immunoprecipitated, and its association with the reporter RNAs was assessed. As shown in Figure 3C, CNBP bound readily to the reporter carrying the WT *RAB13* 3′ UTR but not to the truncated UTR (Figure 3C, left graph), even though equal levels of CNBP were recovered in each case (Figure 3C, right graph). Through *in vivo* imaging, we verified that the two reporters exhibited distinct distributions in the cytoplasm (Figure 3B). The reporter carrying the WT 3′ UTR accumulated in peripheral protrusions, while the one carrying deletion of the GA-rich sequence assumed a more diffuse, perinuclear distribution. Importantly, the reduced association of CNBP with the ΔGA reporter was not due to fact that the mRNA was distributed in the cytoplasm in a way that made it inaccessible to CNBP. In fact, CNBP exhibits a distribution similar to that of the ΔGA reporter, with the bulk of the protein showing a diffuse perinuclear accumulation by immunofluorescence staining (Figure S3). Additionally, distribution of the WT reporter depends on CNBP and KIF1C, as siRNA-mediated knockdown of either factor significantly decreased the peripheral accumulation of the WT reporter (Figures 3D and 3E). By contrast, the distribution of the ΔGA reporter was not affected (Figure 3E), and the distribution of *RHOA* mRNA in the same cells also remained unaffected. Thus, the functional role of CNBP on peripheral mRNA trafficking appears to be mainly mediated through the GA-rich regions. Altogether, these data indicate that CNBP binds to 3′ UTR GA-rich localization sequences and is required for peripheral mRNA targeting to cell protrusions.

### CNBP is required for microtubule-dependent mRNA trafficking and associates with KIF1C

**Figure 4.**
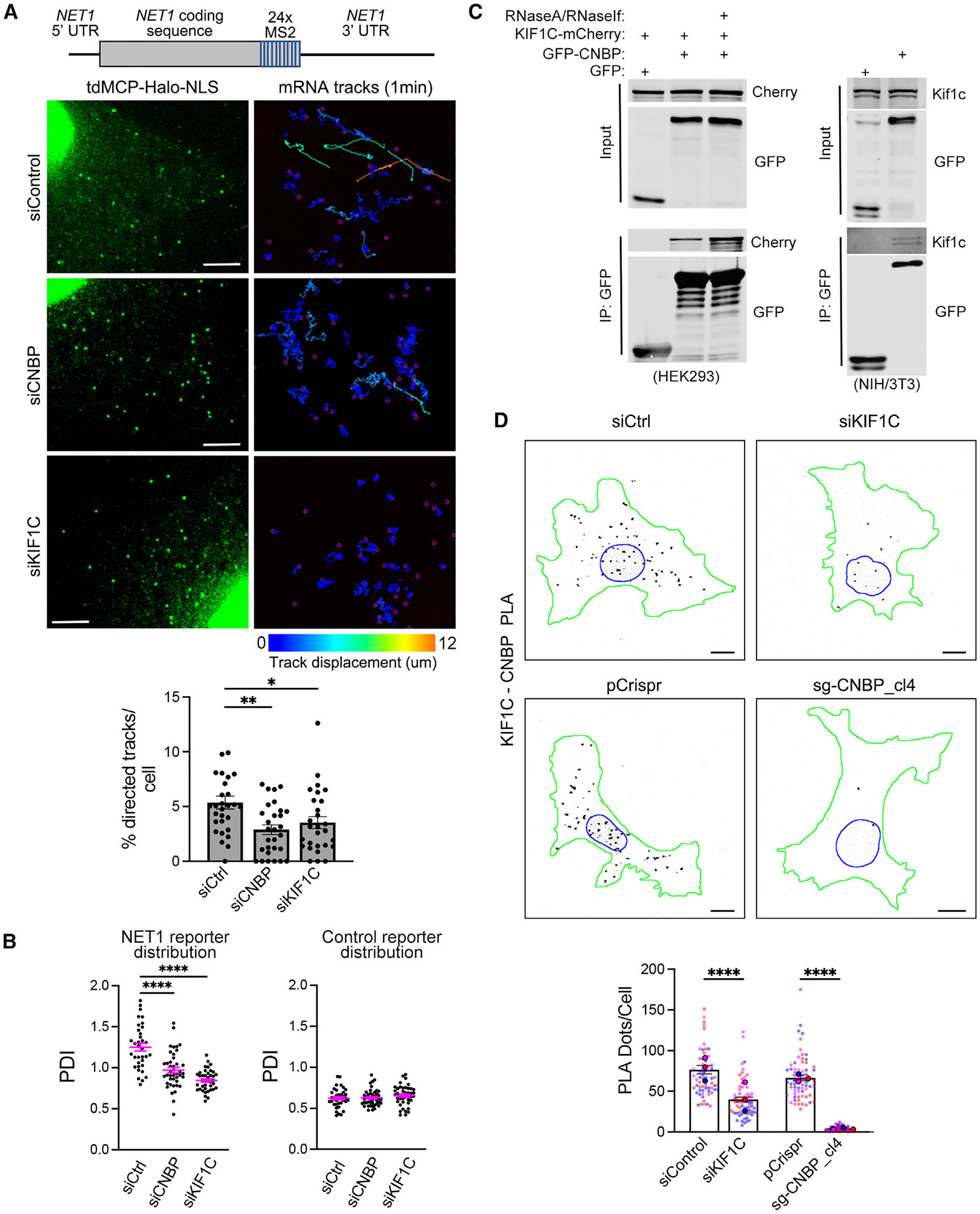
CNBP associates with KIF1C and is required for microtubule-dependent mRNA trafficking (A) Schematic of MS2 reporter RNA containing the NET1 5′ and 3′ UTRs and coding sequence as well as 24 MS2 hairpins. Cells stably expressing the reporter and tdMCP-Halo-NLS were transfected with the indicated siRNAs and high-speed imaging was performed over 1 min to track individual RNA movements. Left: images of magnified areas at the beginning of the time lapse. Scale bar: 4 μm. Right: accumulated tracks over the 1 min imaging period. Tracks are color coded according to total displacement in μm. Bottom graph: percentage of directed tracks per cell following treatment with the indicated siRNAs. *n* = 28–29 cells. *p < 0.05 and **p < 0.01 by Kruskal-Wallis test with Dunn’s multiple comparisons test. (B) PDI quantifications of NET1 reporter distribution, or of a reporter carrying a control 3′ UTR, from cells treated with the indicated siRNAs. *n* = 35–42 cells. ****p < 0.0001 by Kruskal-Wallis test with Dunn’s multiple comparisons test. (C) Representative western blots following GFP-CNBP immunoprecipitation. GFP-CNBP interacts with KIF1C-mCherry in an RNA-independent manner (left, HEK293 cells), as well as with endogenous Kif1c (right, NIH/3T3 cells). (D) Representative images of *in situ* detection of interaction between CNBP and KIF1C by PLA in the indicated CRISPR-edited cell lines or cells treated with the indicated siRNAs. Black dots: PLA signal; blue outline: nuclear boundary; green outline: cell boundary. Scale bars: 10 μm. Bottom graph: quantification of PLA dots per cell. *n* = 65–67 cells in 3 independent experiments. Data points from individual replicates are color coded, and large, outlined color dots indicate the mean of each replicate. Error bars: SEM. ****p < 0.0001 by Kruskal-Wallis test with Dunn’s multiple comparisons test. See also Figure S4 and Videos S1, S2, and S3.

mRNA targeting to cell protrusions additionally depends on the KIF1C kinesin motor.^42^ KIF1C co-traffics with protrusion-targeted mRNAs and is required for their long and directed motions on microtubules.^42^ To determine whether CNBP also affects the microtubule-based transport of protrusion-localized mRNAs, we examined their trafficking upon CNBP knockdown. We have previously reported, using single-molecule mRNA tracking in live cells, that a fraction of mRNAs exhibit long and linear motions over an imaging period of 1 min in duration. These motions depend on microtubules and KIF1C.^42^ For the studies described here, we have constructed an improved MS2-based reporter that incorporates all the regulatory elements of the NET1 transcript: the 5′ and 3′ UTRs as well as the NET1A coding sequence (Figure 4A, schematic). The 3′ UTR of NET1 is sufficient to direct protrusion localization of this reporter. Single-molecule RNA tracking was performed in cells expressing MCP-Halo upon the addition of a fluorescent Halo ligand (Figure 4A; Videos S1, S2, and S3). As reported previously,^42^ during the imaging period, about 5% of tracks per cell were long and directed in control cells and were significantly reduced in cells after the transient siRNA-mediated knockdown of KIF1C. Importantly, we found that CNBP knockdown reduced the fractions of long and directed tracks to a similar extent (Figures 4A and S4). Therefore, both CNBP and KIF1C participate in the microtubule-based trafficking of protrusion-localized mRNAs.

The small fraction of observed actively directed tracks is likely due to the relatively brief 1 min imaging window. To orthogonally assess whether the observed differences in mobility parameters correlate with the overall changes in cytoplasmic RNA distribution, we measured the steady-state distribution of the reporter mRNA using the PDI metric. Indeed, peripheral targeting of the NET1 reporter was significantly reduced upon CNBP or KIF1C knockdown (Figure 4B). This effect was specific for peripherally targeted reporters (carrying either the NET1 or RAB13 UTRs; Figures 3E and 4B, respectively), while the distribution of a non-targeted reporter carrying a control 3′ UTR sequence was not affected upon CNBP or KIF1C knockdown (Figure 4B).

**Figure 5.**
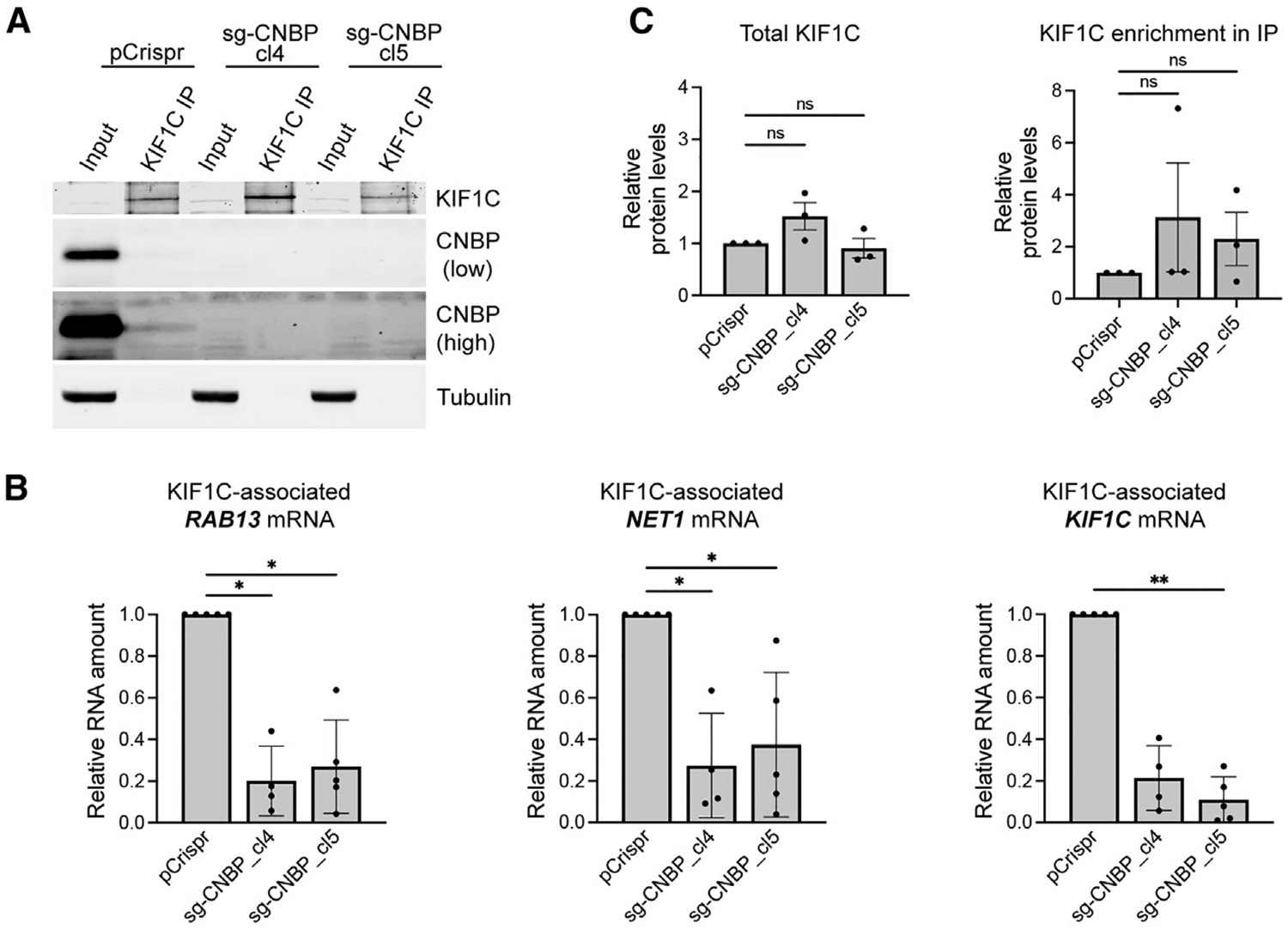
CNBP is required for recruitment of KIF1C to protrusion-localized mRNAs KIF1C was immunoprecipitated from the indicated CRISPR-edited cell lines (pCrispr control or CNBP-knockout clones). (A) Western blot analysis of the indicated proteins from input or immunoprecipitation (IP) samples. CNBP blot is shown with an adjusted contrast to highlight the CNBP protein detected in association with KIF1C (pCrispr-KIF1C IP lane). (B) Amount of indicated mRNAs in KIF1C immunoprecipitates by ddPCR. *n* = 4–5 independent replicates. *p < 0.05 and **p < 0.01 by Kruskal-Wallis test with Dunn’s multiple comparisons test. (C) Quantification of total KIF1C levels and efficiency of KIF1C IP in control or CNBP-knockout cells. Non-significant differences by Kruskal-Wallis test with Dunn’s multiple comparisons test. See also Figure S5.

RBPs can function as adaptors that recruit molecular motors to RNA localization sequences (see introduction). To gain insights into how CNBP participates in the localization mechanism, we sought to determine whether CNBP and KIF1C physically interact. In co-immunoprecipitation experiments, the two proteins readily and specifically interacted when tagged forms were overexpressed in HEK293 cells (Figure 4C, left). This interaction persisted when RNA in the lysate was degraded by RNase treatment (Figure 4C), suggesting a direct physical association of the two proteins rather than an indirect interaction through binding to common RNA molecules. We additionally tested the interaction between endogenous KIF1C and exogenous GFP-tagged CNBP in NIH/3T3 cells and could again detect a specific, albeit less prominent, association (Figure 4C, right). We could also occasionally, but not consistently, observe an interaction when detecting the endogenous proteins (see, for example, Figure 5A). Overall, we conclude that there is a specific, RNA-independent interaction between CNBP and KIF1C and that, under normal expression conditions, likely only a small fraction of each of the two proteins is engaged in a complex in cells.

We, further, alternatively interrogated this association with a proximity ligation amplification (PLA) assay (Figure 4D). Using antibodies that recognize the endogenous proteins, we detected a substantial PLA signal under control conditions (cells stably expressing only Cas9 [pCrispr] or transiently transfected with a control siRNA [siCtrl]). The observed signal specifically reflected *in situ* CNBP-KIF1C complexes since it was significantly reduced in CNBP-knockout cells or upon transient KIF1C knockdown (Figure 4D). Interestingly, PLA dots were distributed throughout the cell, with a small bias around the nucleus, but were noticeably absent from peripheral regions, where protrusion-localized mRNAs accumulate. These results demonstrate that CNBP and KIF1C physically interact in the bulk cytoplasm. They further indicate that their interaction is likely disrupted or altered at peripheral locations.

### CNBP is required for recruitment of KIF1C to protrusion-localized mRNAs

The fact that CNBP interacts both with RNA localization sequences and KIF1C suggested that it might function as an adaptor for the recruitment of KIF1C to protrusion-localized mRNAs. To test this idea, we assessed whether the loss of CNBP would affect the ability of KIF1C to associate with protrusion-localized mRNAs. For this, we immunoprecipitated KIF1C, either from control cells or the two clonal CNBP knockout cell lines (Figure 5A), and quantified the number of associated mRNAs by ddPCR (Figure 5B). Indeed, in the absence of CNBP, KIF1C associated with protrusion-localized mRNAs (*RAB13*, *NET1*, and with its own *KIF1C* mRNA) to a significantly lower degree (Figure 5B). Importantly, this reduction was not due to changes in the overall expression of KIF1C or the efficiency of KIF1C immunoprecipitation (Figure 5C). Conversely, the loss of KIF1C did not affect the binding of CNBP to these mRNAs (Figure S5). If anything, upon KIF1C loss, CNBP-RNA binding appeared slightly increased. This could potentially reflect that RNAs, which are not trafficked efficiently in the absence of the KIF1C,^42^ remain at perinuclear regions where the bulk of CNBP is also found (see above and Figure S3), resulting in an increased CNBP-RNA association. Regardless of this latter possibility, overall, these data show that CNBP binds to protrusion-localized mRNAs independently of KIF1C but is itself required for KIF1C’s association with protrusion-localized mRNAs, consistent with a role for CNBP as an adaptor for motor recruitment.

### A CNBP-KIF1C motor complex is recruited at GA-rich regions of protrusion-localized mRNAs

**Figure 6.**
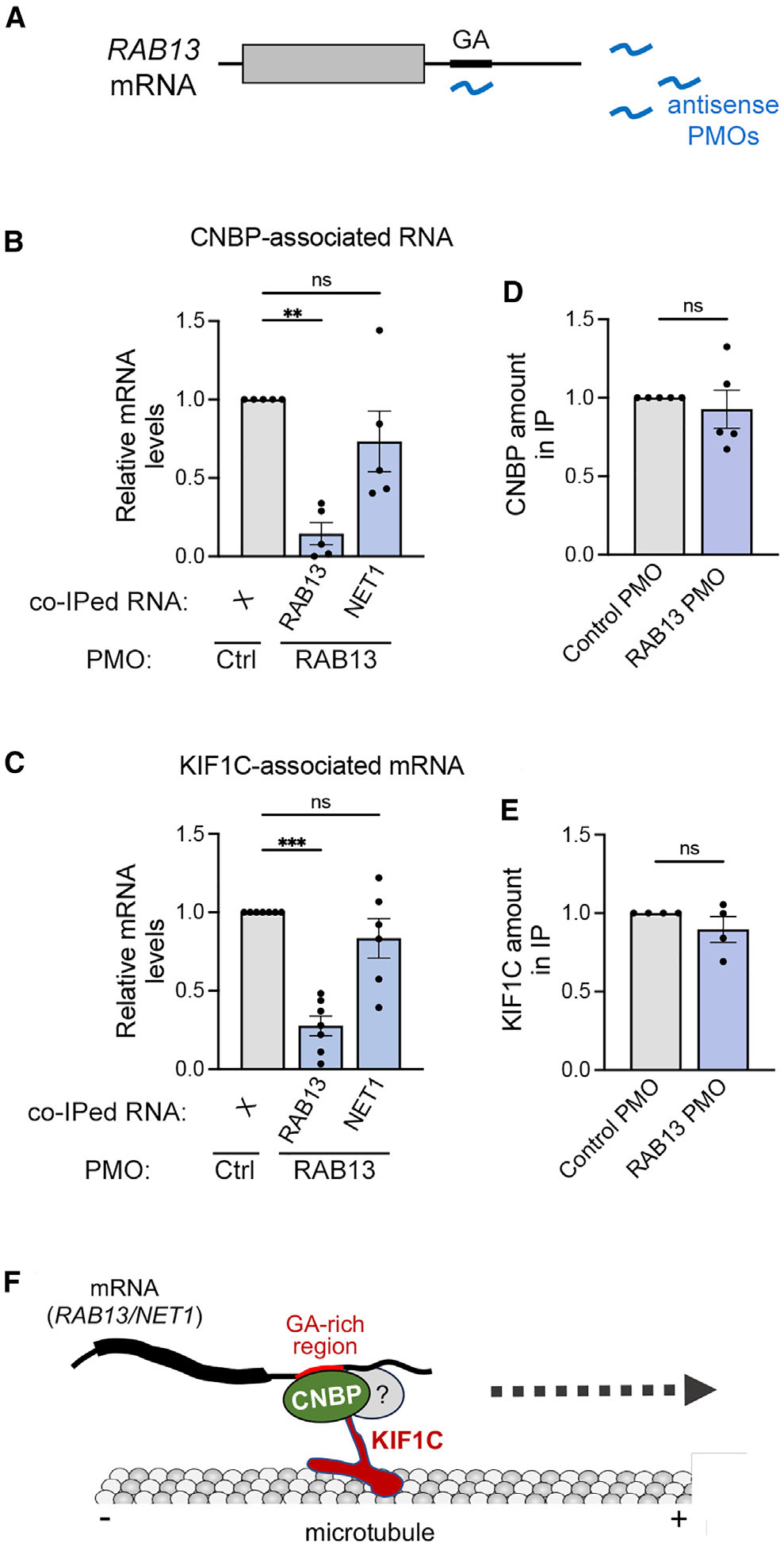
Localization-blocking PMOs against GA-rich regions prevent CNBP and KIF1C binding to target mRNAs (A) Schematic of experimental approach. Antisense PMOs are delivered into cells. PMOs targeting the GA-rich region of the *RAB13* mRNA prevent *RAB13* localization, potentially through interfering with RBP binding. (B and C) CNBP (B) or KIF1C (C) immunoprecipitation to detect amount of associated RNAs after PMO delivery. Note that binding to *RAB13* mRNA is specifically affected upon delivery of PMOs targeting the RAB13 GA-region. *n* = 5 (B) and 7 (C) independent replicates. Error bars: SEM. ***p < 0.001, **p < 0.01, and ns, non-significant, by Kruskal-Wallis test with Dunn’s multiple comparisons test. (D and E) Relative amount of CNBP (D) or KIF1C (E) protein recovered in immunoprecipitates (IPs) by western blot. ns, non-significant, by Wilcoxon matched-pairs signed-rank test. (F) Proposed model for motor-adaptor complex directing mRNA trafficking to cell protrusions. CNBP binds to GA-rich regions within the 3′ UTRs of protrusion-targeted mRNAs and serves as an adaptor for the recruitment of the KIF1C kinesin. Additional factors could participate in this complex. KIF1C subsequently traffics mRNAs on microtubules toward the cell periphery.

The above data suggest that the KIF1C motor is recruited at GA-rich regions of protrusion-localized mRNAs through CNBP. To provide further support for this model, we employed antisense phosphorodiamidate morpholino oligonucleotides (PMOs) that target the GA-rich region of the human *RAB13* mRNA (Figure 6A). When delivered into cells, these antisense oligos specifically disrupt the peripheral localization of the targeted mRNA^36,37^; however, the underlying mechanism of action was unknown. We reasoned that the hybridization of these oligos to GA-rich regions might interfere with CNBP-KIF1C complex recruitment. If this prediction is correct, then it would support the model of motor recruitment at these UTRs and, additionally, provide a molecular understanding of how antisense oligos interfere with protrusion mRNA localization.

To address this, either non-targeting control oligos or oligos targeting the GA-rich region of human *RAB13* mRNA were delivered into cells. CNBP or KIF1C proteins were then immunoprecipitated, and the number of *RAB13* or *NET1* mRNAs associating with each protein was assessed by ddPCR. The delivery of oligos against *RAB13* led to a significant reduction in the amount of *RAB13* mRNA that associated with CNBP (Figure 6B), indicating that CNBP binding is prevented by oligo hybridization to the GA-rich region. Importantly, the amount of *RAB13* mRNA that associated with KIF1C was also significantly reduced (Figure 6C), consistent with the model that KIF1C recruitment relies on CNBP. The observed reduced *RAB13* RNA association with CNBP and KIF1C upon oligo treatment could not be explained by changes in the amount of the corresponding proteins that were immunoprecipitated or expressed in cells (Figures 6D and 6E). Furthermore, the delivery of oligos targeting the GA-rich region of *RAB13* did not affect the binding of CNBP or KIF1C to the *NET1* mRNA, another protrusion-localized mRNA (Figures 6B and 6C). These results are consistent with the previously reported specificity of these oligos, which have been shown to affect only their target mRNA.^36-38^ They further indicate that the CNBP-KIF1C motor complex is recruited independently on different mRNAs. Altogether, these results strongly support a model where GA-rich UTRs provide a platform for the binding of CNBP and the subsequent recruitment of the KIF1C motor for trafficking to cell protrusions (Figure 6F).

## DISCUSSION

RNA trafficking to mammalian cell protrusions depends on 3′ UTR RNA sequences and the KIF1C kinesin.^42^ Here, we have searched for additional components that participate in this transport pathway. We have identified the RBP CNBP as a factor that directly associates with localization sequences in the 3′ UTR of protrusion-localized mRNAs and mediates their peripheral targeting. Our data indicate that CNBP links individual mRNA cargo to the kinesin motor, thus directing their trafficking to cell protrusions. This model is based on the following findings: first, CNBP binds directly to protrusion-localized mRNAs, and binding requires the same GA-rich sequences located in their 3′ UTR that are needed for proper localization. Second, CNBP interacts with KIF1C both physically and functionally. Third, CNBP is required for KIF1C recruitment to mRNA. We cannot exclude that additional factors might participate in KIF1C recruitment or that CNBP has additional roles, such as regulating KIF1C motor activity. Nevertheless, the evidence presented here supports a simple model of an adaptor-motor complex. This work adds to our understanding of the molecular mechanisms connecting mRNAs to transport machinery for long-range movements in the cytoplasm.

CNBP has been described as a nucleic acid (single-stranded DNA [ssDNA] and RNA)-binding protein featuring six to seven tandem CCHC-type zinc knuckle motifs.^46^ An Arg/Gly-rich motif on CNBP is important for binding nucleic acid sequences that are enriched in G nucleotides.^46,47^ Its binding to and unfolding of complex secondary structures, such as G-quadruplexes, has been proposed to facilitate and translation.^46-50^ The work presented here adds to the existing functions of CNBP by describing a novel role in RNA localization by promoting the recruitment of the KIF1C kinesin to RNAs.

We show that CNBP is required for long and linear mRNA movements in the cytoplasm, likely occurring on microtubule tracks. In agreement, we observe that the interaction of CNBP with KIF1C is readily observed by the PLA assay in the bulk cytoplasmic region, where a large fraction of microtubule-dependent trafficking occurs. Interestingly, however, CNBP-KIF1C interaction is not prominently detected in the cell periphery. We have shown previously that protrusion-targeted mRNAs exist in two physical states: as monomers, which comprise a major fraction of the transcript population, and as clusters, which are predominantly located at the tips of retracting protrusions and heterogeneous, composed of multiple mRNA species.^40^ While KIF1C readily accumulates in peripheral RNA clusters,^42^ we do not observe a CNBP-KIF1C association in these regions. We cannot determine whether CNBP persists on mRNAs throughout trafficking or what triggers a change in its interaction with KIF1C. An interesting possibility suggested by our observations is that CNBP dissociates when mRNAs reach the cell periphery and become incorporated into KIF1C-containing clusters.

Afew possibilities could underlie such a switch in KIF1C-RNA association. KIF1C could associate with RNAs through other zinc-finger (ZnF)-containing RBPs, such as MBNL.^43^ Interestingly, the unstructured carboxy-terminal tail of MBNL supports association with membranes,^43^ raising the intriguing possibility that such a potential RBP switch, from CNBP to MBNL, could maintain KIF1C association while further anchoring the RNA to the proximal plasma membrane at peripheral sites. In a different scenario, KIF1C might directly bind to RNA. Indeed, KIF1C can be crosslinked to mRNAs.^51-53^ Additionally, KIF1C contains a C-terminal intrinsically disorder region (IDR) that drives liquid-liquid phase separation (LLPS) at peripheral protrusive regions.^54^ The purified C-terminal KIF1C IDR can bind and recruit RNA in *in vitro* condensates in a sequence-specific manner.^54^ Thus, the increased local KIF1C concentration at the cell periphery could lead to its condensation and direct association with RNA. In this context, it might be relevant that CNBP was recently reported to display antiviral activity against SARS-CoV-2 through binding to the viral RNA and preventing viral RNA and nucleocapsid protein from forming LLPS condensates, which are important for viral replication.^55^ Therefore, one could speculate that CNBP dissociation might be important in allowing KIF1C-RNA condensation at cell protrusions. Delineating how this apparent switch in the mode of KIF1C binding is triggered on a molecular level and what functional role it might serve are interesting future questions.

Another aspect of RNA regulation that differs between the bulk cytoplasm and the cell periphery is translation. Protrusion-targeted mRNAs, when they are in a monomeric form in the bulk cytoplasm, are actively translated and exhibit the same translational output independent of their position in the cytoplasm.^40^ On the other hand, peripheral mRNAs found in condensate-like clusters appear translationally repressed, as evidenced by their association with fewer nascent polypeptides.^40^ Given that CNBP has been shown to promote translation by binding G-rich elements,^46-48^ we envision that CNBP might additionally participate in translational regulation. In this way, CNBP dissociation from mRNAs at the periphery might lead to translational down-regulation and thus coordinate KIF1C-dependent RNA condensation with translational repression. Therefore, CNBP might have a dual role in the regulation of protrusion-localized mRNAs, serving as a kinesin adaptor involved in their trafficking and as a spatial regulator of their translation.

Finally, alterations in the CNBP locus have been associated with myotonic dystrophy (dystrophia myotonica [DM]) type 2 (DM2).^56^ Specifically, an expansion of hundreds to thousands of CCTG repeats within intron 1 of the CNBP gene results in intron retention and is thought to be the cause of the condition.^56^ Similarly, a CTG repeat expansion in the 3′ UTR of the DMPK gene has been linked to myotonic dystrophy type 1 through the production of a toxic transcript that forms foci in the nucleus and sequesters the MBNL family of RBPs.^57,58^ In turn, functional compromise of MBNL results in dysregulation of the metabolism of several mRNAs. It has been proposed that a similar indirect, MBNL-dependent mechanism can underlie at least part of the DM2 phenotype. However, a direct role of CNBP cannot be excluded. Although the intronic repeat expansion in CNBP does not alter the amount of transcript produced or its export to the cytoplasm,^59^ reduced levels of protein expression have been reported.^49,60^ Importantly, heterozygous or homozygous CNBP-knockout mouse models recapitulate a variety of muscle features observed in DM2,^61,62^ supporting the idea that at least some of the DM2 defects could be a direct consequence of compromised CNBP protein expression. In light of the roles of both CNBP and MBNL as KIF1C adaptors and the dual role of CNBP as a translation regulator and a kinesin adaptor discussed here, this work provides the basis for interesting hypotheses toward understanding disease mechanisms.

### Limitations of the study

Our data show that CNBP associates with KIF1C and is required for KIF1C recruitment on mRNAs. However, we cannot currently conclude whether CNBP is by itself sufficient for KIF1C recruitment. It is possible that additional factors participate together with CNBP and contribute to motor binding. The potential existence of additional KIF1C adaptors or additional modes of KIF1C-RNA binding is also suggested by our observation that CNBP does not interact with KIF1C at the peripheral edges of cells, even though KIF1C has been shown to still colocalize with mRNAs at these areas. As discussed above, this suggests a change in KIF1C-RNA interactions. We cannot currently determine the exact cellular location where the CNBP-KIF1C association is disrupted or what signal might trigger such a change. An alternative possibility could be that CNBP is only involved in loading KIF1C on mRNAs and does not persist during trafficking on microtubules. Further work would be necessary to clarify these aspects of the trafficking mechanism.

## STAR★METHODS

### EXPERIMENTAL MODEL AND STUDY PARTICIPANT DETAILS

#### Cell culture

MDA-MB-231 cells were obtained from ATCC (cat # HTB-26) and cultured in Leibovitz’s L-15 medium (Invitrogen cat# 11415064) supplemented with 10% FBS (or Tet-approved FBS for cells expressing inducible reporter RNAs) and 1% Penicillin-Streptomycin at 37°C in atmospheric air in a humidified cell culture incubator. NIH/3T3 mouse fibroblast cells were cultured in DMEM (Invitrogen cat# 1995073) supplemented with 10% calf serum, sodium pyruvate and 1% Penicillin-Streptomycin at 37°C and 5% CO_2_. Cells were passaged by trypsinization using 0.05% trypsin (Invitrogen cat# 25300120). Cells used in this study have tested negative for mycoplasma.

#### Cell lines

To generate stable cell lines expressing RNA reporters, MDA-MB-231 cells were infected with lentivirus expressing stdMCP-stdHalo (Addgene #104999, modified to remove the Kozak sequence and ATG initiating codon from the sequence of the first HaloTag). A uniformly expressing cell population was selected by fluorescence-activated cell sorting. This population was then infected with pInducer20-based constructs expressing β-globin followed by 18xMS2 binding sites and either the wild type human RAB13 3′UTR (accession#: GenBank: NM_002870.5) or a deletion mutant lacking a GA-rich region of the RAB13 3′UTR (corresponding to nucleotides 202–254). NIH/3T3 cells were also infected with stdMCP-stdHalo and with a pInducer20-based construct expressing the NET1A 5′ UTR, coding sequence and 3′UTR together with 24xMS2v7 repeats after the coding sequence. Stably expressing lines were selected with Geneticin (Thermo Fisher Scientific). Expression of the reporters was induced by addition of 1 μg/mL doxycycline approximately 3–4 h before imaging.

To generate CRISPR edited lines, NIH/3T3 or MDA-MB-231 cells were infected with pLentiCRISPRv2 expressing appropriate guide RNAs and selected with puromycin. Where indicated individual clonal cell lines were isolated by limited dilution.

### METHOD DETAILS

#### Plasmid constructs and lentivirus production

To generate templates for *in vitro* transcription, inserts containing a T7 promoter, and the indicated UTRs followed by a BoxB sequence were cloned into HindIII and EcoRI sites of pTZ19R (exact sequences used are indicated in Table S2). Plasmids linearized with EcoRI were used as templates for *in vitro* transcription.

To express EGFP-tagged CNBP, a lentiviral plasmid that contained the human CNBP CDS (GenBank: NM_001127192.2) with an N-terminal EGFP tag driven by the SV40 promoter and a neomycin resistance gene driven by the CMV promoter was purchased from VectorBuilder. To express mCherry-tagged KIF1C, an expression vector containing the human KIF1C CDS with a C-terminal mCherry tag was purchased from Addgene (#130978). These constructs were used in transient transfections using PolyJet *In Vitro* DNA Transfection Reagent (SignaGen).

To generate lentiviral plasmids expressing Cas9 together with single guide RNAs, the pLentiCRISPRv2 plasmid was used (Addgene #52961). Target sequences of cloned sgRNAs were as follows: mCNBP-Ex3: TGCCAAGGATTGTGATCTGC; mHNRNPA2B1-Ex3: TCTCTTGCTACAGCACGTTT; mHNRNPH1-Ex3: TCAACAAAAGCCTCGCCACT; mHNRNPH2-aa19: TCCTCGGCTGAGCA GGACCA; mHNRNPH2-aa50: CGTTTCATCTACACCAGAGA (targets both hnRNPH1 and hnRNPH2); hsCNBP-Ex2-1: GTGTG GACGATCTGGCCACT. sgRNA sequences were cloned onto BsmBI sites of pLentiCRISPRv2 and plasmids were used for lentivirus production.

Lentiviruses were produced in HEK293T cells. The cells were transfected with the lentiviral vectors together with the pMD2.G and psPAX2 packaging plasmids using PolyJet *In Vitro* DNA Transfection Reagent (SignaGen) for 48 h. Harvested virus was precipitated with polyethylene glycol overnight at 4°C.

#### *In vitro* transcription and λN-GST pulldown

*In vitro* transcriptions were carried out from linearized plasmid templates (4 ugr in 50 μl) using T7 Polymerase HC (Promega, cat# P2075) in transcription optimized buffer (Promega) with 20mM DTT, 10mM MgCl_2_, 4mM NTPs, 40U RNasin Plus (Promega, cat# N2615) and 0.1% Triton X-100. The reaction was incubated at 37°C for 3.5hrs.

For λN-GST pulldown, *in vitro* transcription reactions were incubated with 45ug purified λN-GST protein for 15 min at room temperature. The mix was incubated with 15uL Glutathione magnetic beads (Invitrogen, cat #78602) for 20 min at room temperature with shaking, and the beads and bound RNA were washed with lysis buffer (50mM Tris-Cl pH 7.5, 0.5% Triton X-100, 100mM NaCl, 2.5mM MgCl_2_) and further blocked with lysis buffer containing E. coli tRNA, BSA (Sigma, cat#10711454001), salmon sperm DNA (Invitrogen, cat# 15632011) and glycogen (Invitrogen, cat# AM9510) for 30–60 min at 4°C. Cell extracts from MDA-MB-231 or NIH/3T3 cells were prepared by lysing in lysis buffer with Halt protease inhibitor (Invitrogen, cat #78444) and RNase inhibitor (Promega, cat# N2615), brief sonication and clearing at 10,000xg for 10 min at 4°C. The GSH beads and bound RNA were incubated with extract from 5 to 10×10^6^ cells at 4°C for 1 h with rotation and washed first with lysis buffer and then with PBS pH 8.0. Bound material was eluted in 50ul of 40mM reduced L-Glutathione in PBS pH 8.0. Bound RNA was analyzed on Agilent Tapestation, and bound protein by Silver staining, Western blot or mass spectrometry.

#### Immunoprecipitation

For KIF1C immunoprecipitation followed by RNA isolation, cells were lysed with a buffer containing 10mM Tris-Cl pH 7.4, 100mM NaCl, 2.5mM MgCl_2_, 0.5% Triton X-100, Halt protease and phosphatase inhibitor cocktail (Invitrogen, cat #78444) and RNase inhibitor (Promega, cat# N2615). Lysates were sonicated (setting 2, 2 × 5sec, Misonix Inc. Sonicator XL), cleared by centrifugation at 14,000xg for 10 min at 4°C, and mixed with Protein G Dynabeads (Invitrogen, cat #100004D) pre-bound with 1ug anti-KIF1C antibody (Bethyl cat# A301-070A) and pre-blocked with E. coli tRNA, salmon sperm DNA (Invitrogen, cat# 15632011), BSA (Sigma, cat#10711454001), and glycogen (Invitrogen, cat# AM9510). Lysate and bead mix was incubated for 1.5 h at 4°C. Bound material was eluted with lysis buffer containing 1% SDS and processed for protein or RNA analysis.

For CNBP immunoprecipitation followed by RNA isolation, cells were crosslinked with 0.3% paraformaldehyde (Electron Microscopy Sciences, cat #15710) in PBS for 10 min at room temperature. Crosslinking was stopped by addition of 250mM glycine pH 7.0, and cells were lysed in RIPA buffer (50mM Tris-Cl pH 7.4, 150mM NaCl, 1% Triton X-100, 0.5% Na-deoxycholate, 0.1% SDS) with Halt protease and phosphatase inhibitor cocktail (Invitrogen, cat #78444) and RNase inhibitor (Promega, cat# N2615). Lysates were sonicated (setting 4.5, 5 Ó 15sec, Misonix Inc. Sonicator XL), cleared by centrifugation at 14,000xg for 10 min at 4°C, and mixed with Protein G Dynabeads pre-bound with 2ug anti-CNBP antibody (Proteintech cat# 67109) and pre-blocked with E. coli tRNA, salmon sperm DNA, BSA, and glycogen. Lysate and bead mix was incubated for 3 h at 4°C. Bound material was eluted with a buffer containing 100mM Tris-Cl pH 6.8, 5mM EDTA, 10mM DTT, 1% SDS, incubated at 70°C for 50 min, and processed for protein analysis. For RNA analysis samples were further digested with proteinase K at 37°C for 30 min before Trizol extraction.

For immunoprecipitation of EGFP-tagged CNBP, cells transiently expressing EGFP-CNBP or EGFP alone or in combination with KIF1C-mCherry were lysed with a buffer containing 50mM Tris pH 7.4, 1% NP-40, 150mM NaCl, 10mM MgCl_2_, 10% glycerol and Halt protease and phosphatase inhibitor cocktail (ThermoFisher). Lysates were cleared by centrifugation at 4°C and added to GFP-Trap Magnetic agarose beads (Chromotek, cat# gtma-10) for 1 h at 4°C. To degrade RNA, cleared lysates were incubated with RNaseA (50 μg/ml; ThermoFisher) and RNase If (300 μg/ml; New England Biolabs) for 30 min at RT, re-centrifuged briefly and added to GFP-Trap beads. Immobilized complexes were eluted with Laemmli’s buffer and analyzed by SDS-PAGE and immunoblotting.

#### siRNA and morpholino transfection

For knockdown experiments, 40 pmol of siRNA were transfected into cells by Lipofectamine RNAiMAX (ThermoFisher, cat# 13778-150) according to the manufacturer’s protocol. Cells were assayed 72 h post-transfection. The following siRNAs were used: AllStars negative control (Qiagen cat# 1027281), si-Mm-Cnbp #6 (Qiagen cat# SI02672313; target sequence: 5′- CAGCAAGACAAG TGAAGTCAA -3′), si-Mm-hnRNPA2B1 #3 (Qiagen cat# SI00210672; target sequence: 5′- AAGGCATTGTCTAGACAAGAA -3′), si-Mm-Hnrnph1 #4 (Qiagen cat# SI01068403; target sequence: 5′- TAGGAGCTGCGTCTACAATTA -3′), si-Mm-hnrnph2 #1 (Qiagen cat# SI01068452; target sequence: 5′- ATGTTGTAGGAGTGTACTTAA -3′)

Antisense morpholino oligonucleotides were synthesized by GeneTools, LLC and delivered into cells using EndoPorter(PEG) (GeneTools, LLC). A combination of two oligos were used for RAB13 and NET1, at a final concentration of 20 μM. Sequences used are as follows:

Control: 5′-CCTCTTACCTCAGTTACAATTTATA-3’;

RAB13: 5′ -TCTTTCACTTCCTCAATTCATTCCT-3′ and

5′-CCTTCCTTTCCTCCTCCCTCTCTTC-3’;

NET1: 5′-TCCCTCTTGCATTTCAGACAACACT-3′ and

5′-GACAAAACTACTCTCTTTTCCTCTC-3’. Cells were assayed 72 h post-transfection.

#### Western blot

The following primary antibodies were used: rabbit polyclonal anti-hnRNPH1 (Bethyl, cat# A300-511A; 1:10,000), rabbit monoclonal anti-hnRNPH2 (Abcam, cat# ab179439; 1:1,000), rabbit polyclonal hnRNPF (Abcam, cat# ab50982; 1:1,000), mouse monoclonal hnRNPA2 (Santa Cruz, cat# sc-53531; 1:1,000), mouse monoclonal anti-CNBP (Proteintech, cat# 67109; 1:3,000), rabbit polyclonal anti-CNBP (ThermoFisher, cat# PA5-35241; 1:1,000), rabbit polyclonal anti-KIF1C (Bethyl, cat# A301-070A; 1:2,000), rabbit polyclonal anti-KIF1C (Proteintech, cat# 12760-1-AP; 1:1,000), mouse monoclonal hnRNPK (Santa Cruz, cat# sc-28380), rabbit monoclonal anti-GAPDH (Proteintech, cat# 2118), rabbit polyclonal anti-GFP (Invitrogen, cat# A-11122; 1:2,000) and rabbit polyclonal anti-Cherry (Abcam, cat# ab183628; 1:5,000). Anti-rabbit and anti-mouse secondary antibodies from Li-Cor were used at 1:10,000. Membranes were scanned using an Odyssey fluorescent scanner (Li-Cor) and bands were quantified using ImageStudioLite (Li-Cor).

#### Mass spectrometry

Proteins were denatured by addition of 8M urea in 100 mM ammonium bicarbonate and then heating at 37°C for 15 min. They were then reduced by reaction with 10 mM DTT for 1 h at 37°C and reduced by reaction with 50 mM iodoacetamide for 30 min at room temperature. The urea concentration was reduced to 1 M and proteins trypsin digested overnight at 37°C. Following digestion, the peptides were desalted on a C18 spin desalting column and dried by lyophilization. Dried peptides were resuspended in 5% acetonitrile, 0.05% TFA in water for mass spectrometry analysis on an Obitrap Fusion Tribrid (Thermo) mass spectrometer. The peptides were separated on a 75 μm × 15 cm, 3 μm Acclaim PepMap reverse phase column (Thermo) at 300 nL/min using an UltiMate 3000 RSLCnano HPLC (Thermo) and eluted directly into the mass spectrometer. For analysis, parent full-scan mass spectra collected in the Orbitrap mass analyzer set to acquire data at 120,000 FWHM resolution and HCD fragment ions detected in the ion trap. Proteome Discoverer 2.0 (Thermo) was used to search the data against the murine database from Uniprot using SequestHT. The search was limited to tryptic peptides, with maximally two missed cleavages allowed. Cysteine carbamidomethylation was set as a fixed modification, with methionine oxidation as a variable modification. The precursor mass tolerance was 10 ppm, and the fragment mass tolerance was 0.8 Da. The Percolator node was used to score and rank peptide matches using a 1% false discovery rate.

#### RNA isolation and ddPCR

RNA was extracted using Trizol LS reagent (Invitrogen, cat # 10296028) according to the manufacturer’s instructions. Prior to extraction an equal amount of exogenous spike RNA (*in vitro* transcribed beta-globin or GFP RNA) was added to each sample to correct for differences in recovery during the process. Isolated RNA was treated with RQ1 DNAse (Promega, cat #M6101) for 30 min at 37°C and purified again with Trizol. RNA was reverse transcribed using the iScript cDNA Synthesis Kit (Bio-Rad, cat# 1708891). For ddPCR, cDNA samples were analyzed using the ddPCR EvaGreen Supermix (Bio-Rad, cat. no. 186–4034). Droplets were generated using the Automated Droplet Generator (Bio-Rad, cat no. 186–4101), PCR amplification was performed on a C1000 Touch Thermal Cycler (Bio-Rad, cat no. 185–1197) and droplet reading was done with the QX 200 Droplet reader (Bio-Rad, cat no. 186–4003) and QuantaSoft software (Bio-rad). Specificity of primers pairs in detecting RAB13, NET1, KIF1C, beta-globin and GFP RNAs was verified by comparing with RNA from knockdown cells.

#### RNA fluorescence in situ hybridization (FISH)

MDA-MB-231 or NIH/3T3 cells were plated on either collagen IV (Sigma, cat# C5533; 10ug/mL) or fibronectin (Sigma, cat# F1141; 5ug/mL) coated coverslips, respectively, for 2hrs and fixed with 4% paraformaldehyde for 20 min at RT. FISH was performed using the ViewRNA ISH Cell Assay kit (ThermoFisher, cat# QVC0001) according to the manufacturer’s protocol. The following probes were used in this study: mouse Rab13 (cat# VB1-14374-01), mouse Net1 (cat# VB1-3034209-01), mouse Cyb5r3 (cat# VB1-18647-01), mouse Ddr2 (cat# VB1-14375-01), human RAB13 (cat# VA1-12225-06), human NET1 (cat# VA1-20646-01), human PKP4 (cat# VA1-12406-01), human RHOA (cat# VA6-14829-01), human HBB (cat# VA6-17839-01), human TRAK2 (cat# VA1-3011278-01), human RPS20 (cat# VA1-16561-01). HCS Green cell mask (Invitrogen, cat# H32714) was used to identify the cell border and samples were mounted in ProLong Gold antifade with DAPI (Invitrogen, cat# P36931).

#### Proximity ligation assay (PLA)

MDA-MB-231 cells plated on collagen IV-coated (10ug/mL) coverslips were washed with PBS and fixed for 15 min at RT with 4% paraformaldehyde then permeabilized for 5 min at RT with 0.2% Triton X-100. The DuoLink *In Situ* Red Kit (Sigma, cat #DUO92008) was used for PLA and the manufacturer’s protocol was followed. Briefly, the cells were blocked using the provided blocking buffer at 37°C for 1hr in a humidified chamber. Primary antibodies were diluted in the provided DuoLink antibody diluent and incubated on the cells for 1.5 hrs at RT in a humidified chamber. The following primary antibodies were used: rabbit anti-KIF1C (Proteintech, cat# 12760-1-AP; 1:100), mouse anti-CNBP (Proteintech, cat# 67109-1-IG; 1:500). After washing, the PLA probes supplied with the kit were used at a 1:10 dilution in the antibody diluent and incubated for 1 h at 37°C. Ligation was performed for 30 min at 37°C then amplification was performed for 100 min at 37°C. The cells were washed and fixed again in 4% paraformaldehyde in PBS for 10 min at RT then stained with Alexa Fluor 488 Phalloidin (Invitrogen, cat# A12379; 1:500) in blocking buffer for 20 min at RT. Coverslips were mounted in the provided DuoLink mounting medium with DAPI and kept at 4°C in the dark until imaging the next day.

### MICROSCOPY AND IMAGE ANALYSIS

RNA FISH and PLA experiments were imaged on a Leica SP8 confocal microscope with an HC PL APO 63x oil immersion objective. Z-stacks through the cell volume were obtained and maximum intensity projections were used for all analysis. For PLA, an ImageJ script based on the Analyze Particles plugin was used to calculate the number of PLA dots present within cells. For FISH images, calculation of the PDI index was performed using a previously published custom MATLAB script.^45^ The PDI index is an intensity weighted measure of the distribution of an RNA population in an individual cell relative to the center of the nucleus. It is also normalized for the size and morphology of each cell on a 2D plane, so that a value above 1 reflects a more peripheral distribution, 1 reflects a diffuse random distribution, while a value below 1 denotes a perinuclear accumulation. We note, however, that mesenchymal cells such as the ones used in this study, due to their spindle shape, have a larger fraction of their volume distributed centrally compared to their peripheral protrusions which are thinner. Thus, in the 2D z-projected images used for PDI analysis, a larger volume fraction is projected on the same 2D area in central versus peripheral areas. Consequently, an mRNA that is diffuse in the cell volume would exhibit a PDI lower than 1. PDI values of 1 or higher indicate a preferential distribution toward the periphery.

Live imaging of cells expressing single-molecule RNA reporters was done using a Nikon Eclipse Ti2-E inverted microscope, equipped with a motorized stage, a Yokogawa CSU-X1 spinning disk confocal scanner unit, and operated using NIS-elements software. Acquisitions were performed using an Apochromat TIRF 1003 oil immersion objective (N.A. 1.49, W.D. 0.12 mm, F.O.V. 22 mm) and Hamamatsu ORCA-Fusion BT Gen III back-illuminated sCMOS cameras. Constant 37°C temperature and 5% CO_2_ were maintained using a Tokai Hit incubation system. To label MCP-Halo proteins, cells were supplemented with 200nM of JFX554 HaloTag ligand, obtained from Janelia Research Campus for 2 h. The medium was then replaced, and 1 mg/mL doxycycline was added to induce expression of reporter mRNAs for 3 h. Cells were plated on fibronectin (5 mg/mL)-coated 35 mm glass bottom dishes for ~1 h, and samples were excited using a 488 nm (20mw) laser line and imaged at a rate of 6.66 fps for 60sec.

Single molecule RNA tracking was performed using TrackMate plugin in ImageJ/Fiji. For every cell, all tracks lasting for >2.5 s (ca. 17 consecutive frames) were used for analysis. Values of ‘Track displacement’, ‘Linearity of forward progression’ and ‘track duration’ were extracted and plotted. ‘Track displacement’ is defined as the distance from the first to the last spot of the track. ‘Linearity of forward progression’ is the mean straight line speed divided by the mean speed; where mean straight line speed is defined as the net displacement divided by the total track time. The thresholds used to filter tracks of molecules undergoing directed movement were based on Pichon et al.^42^

### QUANTIFICATION AND STATISTICAL ANALYSIS

All statistical analysis was performed using GraphPad Prism software using the statistical tests indicated within the text and figure legends. Normally distributed datasets were analyzed using parametric statistical tests. Datasets deviating from a normal distribution were analyzed using non-parametric tests. Follow up tests were included, as appropriate, to adjust for multiple comparisons. The following tests were performed: Student’s t-test, Wilcoxon matched-pairs signed rank test, Kruskal-Wallis test with Dunn’s multiple comparisons test. The sample size and specific test used can be found in each figure legend, and *p*-values are indicated as asterisks: *<0.5, **<0.01, ***<0.001, ****<0.0001.

## Supplementary Material

1

2

3

4

5

## Figures and Tables

**Table:** KEY RESOURCES TABLE

REAGENT or RESOURCE	SOURCE	IDENTIFIER
Antibodies		
rabbit polyclonal anti-hnRNPH1	Bethyl	cat# A300-511A; RRID: AB_203269
rabbit monoclonal anti-hnRNPH2	Abcam	cat# ab179439; RRID: AB_3099469
rabbit polyclonal anti-hnRNPF/H	Abcam	cat# ab50982; RRID: AB_880477
mouse monoclonal anti-hnRNPA2	Santa Cruz	cat# sc-53531; RRID: AB_2248245
mouse monoclonal anti-CNBP	Proteintech	Cat# 67109; RRID: AB_2882413
rabbit polyclonal anti-CNBP	ThermoFisher	cat# PA5-35241; RRID: AB_2552551
rabbit polyclonal anti-KIF1C	Bethyl	cat# A301-070A; RRID: AB_873064
rabbit polyclonal anti-KIF1C	Proteintech	cat# 12760-1-AP; RRID: AB_2131422
mouse monoclonal anti-hnRNPK	Santa Cruz	cat# sc-28380; RRID: AB_627734
rabbit monoclonal anti-GAPDH	Proteintech	cat# 2118; RRID: AB_561053
rabbit polyclonal anti-GFP	Invitrogen	cat# A-11122; RRID: AB_221569
rabbit polyclonal anti-Cherry	Abcam	cat# ab183628; RRID: AB_2650480
Chemicals, peptides, and recombinant proteins		
DMEM media	Invitrogen	cat# 1995073
Trypsin 0.05%	Invitrogen	cat# 25300120
Geneticin	Invitrogen	cat# 10131035
PolyJet *In Vitro* DNA Transfection Reagent	SignaGen	cat# SL100688
T7 Polymerase HC	Promega	cat# P2075
Glutathione Magnetic Beads	Invitrogen	cat# 78602
BSA	Sigma	cat# 10711454001
Salmon sperm DNA	Invitrogen	cat# 15632011
Glycogen	Invitrogen	cat# AM9510
Halt Protease Inhibitor	Invitrogen	cat# 78444
RNase Inhibitor	Promega	cat# N2615
Protein G Dynabeads	Invitrogen	cat# 100004D
GFP-Trap magnetic agarose beads	Chromotek	cat# gtma-10
16% Paraformaldehyde	Electron Microscopy Sciences	cat# 15710
Lipofectamine RNAiMAX	ThermoFisher	cat# 13778-150
EndoPorter (PEG)	GeneTools LLC	cat# N/A
Trizol LS reagent	Invitrogen	cat# 10296028
RQ1 DNAse	Promega	cat# M6101
iScript cDNA Synthesis Kit	Bio-Rad	cat# 1708891
Collagen IV	Sigma	cat# C5533
Fibronectin	Sigma	cat# F1141
ViewRNA ISH Cell Assay Kit	ThermoFisher	cat# QVC0001
ProLong Gold antifade with DAPI	Invitrogen	cat# P36931
DuoLink *In Situ* Red Kit	Sigma	cat# DUO92008
Alexa Fluor 488 Phalloidin	Invitrogen	cat# A12379
RNA FISH Probe - mouse RAB13	Invitrogen	cat# VB1-14374-01
RNA FISH probe - mouse NET1	Invitrogen	cat# VB1-3034209-01
RNA FISH probe - mouse CYB5R3	Invitrogen	cat# VB1-18647-01
RNA FISH probe - mouse DDR2	Invitrogen	cat# VB1-14375-01
RNA FISH probe - human RAB13	Invitrogen	cat# VA1-12225-06
RNA FISH probe - human NET1	Invitrogen	cat# VA1-20646-01
RNA FISH probe - human PKP4	Invitrogen	cat# VA1-12406-01
RNA FISH probe - human TRAK2	Invitrogen	cat# VA1-3011278-01
RNA FISH probe - human RHOA	Invitrogen	cat# VA6-14829-01
RNA FISH probe - human RPS20	Invitrogen	cat# VA1-16561-01
RNA FISH probe - human HBB	Invitrogen	cat# VA6-17839-01
HCS Green Cell Mask	Invitrogen	cat# H32714
Experimental models: Cell lines		
MDA-MB-231	ATCC	cat# HTB-26
NIH/3T3	ATCC	cat# CRL-1658
NIH/3T3 + NLS-HA-stdMCP-stdHalo + scFv-GCN4-sfGFP + pIND20-hNet1A 5UTR-24xGCN4-hNet1A CDS-24xMS2v7_hNet1 3UTR	This study	N/A
MDA-MB-231 + NLS-HA-stdMCP-Halo + pIND20-beta-globin CDS-18xMS2_hRAB13 UTR (WT)	This study	N/A
MDA-MB-231 + NLS-HA-stdMCP-Halo + pIND20-beta-globin CDS-18xMS2_hRAB13 UTR (deltaGA)	This study	N/A
NIH/3T3 + NLS-HA-stdMCP-Halo + pIND20-beta-globin CDS-18xMS2_mNet1 UTR (WT)	This study	N/A
NIH/3T3 + NLS-HA-stdMCP-Halo + pIND20-beta-globin CDS-18xMS2_control UTR (MCS)	This study	N/A
Oligonucleotides		
AllStars negative control	Qiagen	cat# 1027281
si-Mm-CNBP #6	Qiagen	cat# SI02672313
si-Mm-hnRNPA2B1 #3	Qiagen	cat# SI00210672
si-Mm-HnRNPh1 #4	Qiagen	cat# SI01068403
si-Mm-hnRNPh2 #1	Qiagen	cat# SI01068452
Control morpholino	GeneTools LLC	cat# N/A
NET1 morpholino	GeneTools LLC	Cat# N/A
RAB13 morpholino	GeneTools LLC	Cat# N/A
Recombinant DNA		
pLentiCRISPRv2	Addgene	cat# 52961
stdMCP-stdHalo	Addgene	cat# 104999
KIF1C-mCherry	Addgene	cat# 130978
Software and algorithms		
ImageStudioLite	Li-Cor	cat# N/A
Proteome Discoverer 2.0	ThermoFisher	cat# N/A
QuantaSoft software	Bio-Rad	cat# N/A
PDI Analysis MATLAB Script	Stueland et al.^45^	cat# N/A
ImageJ/Fiji	NIH	cat# N/A
LAS X Software	Leica Microsystems	cat# N/A
NIS-Elements Software	Nikon	cat# N/A
Prism software	GraphPad	cat# N/A

## Highlights

- The RNA-binding protein CNBP is needed for targeting of mRNAs to cell protrusions
- CNBP binds directly to protrusion-localized mRNAs through GA-rich regions
- CNBP associates with the KIF1C kinesin
- CNBP is required for recruitment of KIF1C to mRNAs and trafficking on microtubules
