# Supplementary material for: A KIF1C-CNBP motor-adaptor complex for trafficking mRNAs to cell protrusions
Source: Cell Rep. Author manuscript; Available in PMC 2025 Apr 16. (PMC12002053; doi:10.1016/j.celrep.2025.115346)
Supplement: 1 [file NIHMS2069109-supplement-1.pdf]

Cell Reports, Volume *44*

Supplemental information

A KIF1C-CNBP motor-adaptor complex

for trafficking mRNAs to cell protrusions

Konstadinos Moissoglu, Tianhong Wang, Alexander N. Gasparski, Michael Stueland, Elliott L. Paine, Lisa M. Jenkins, and Stavroula Mili

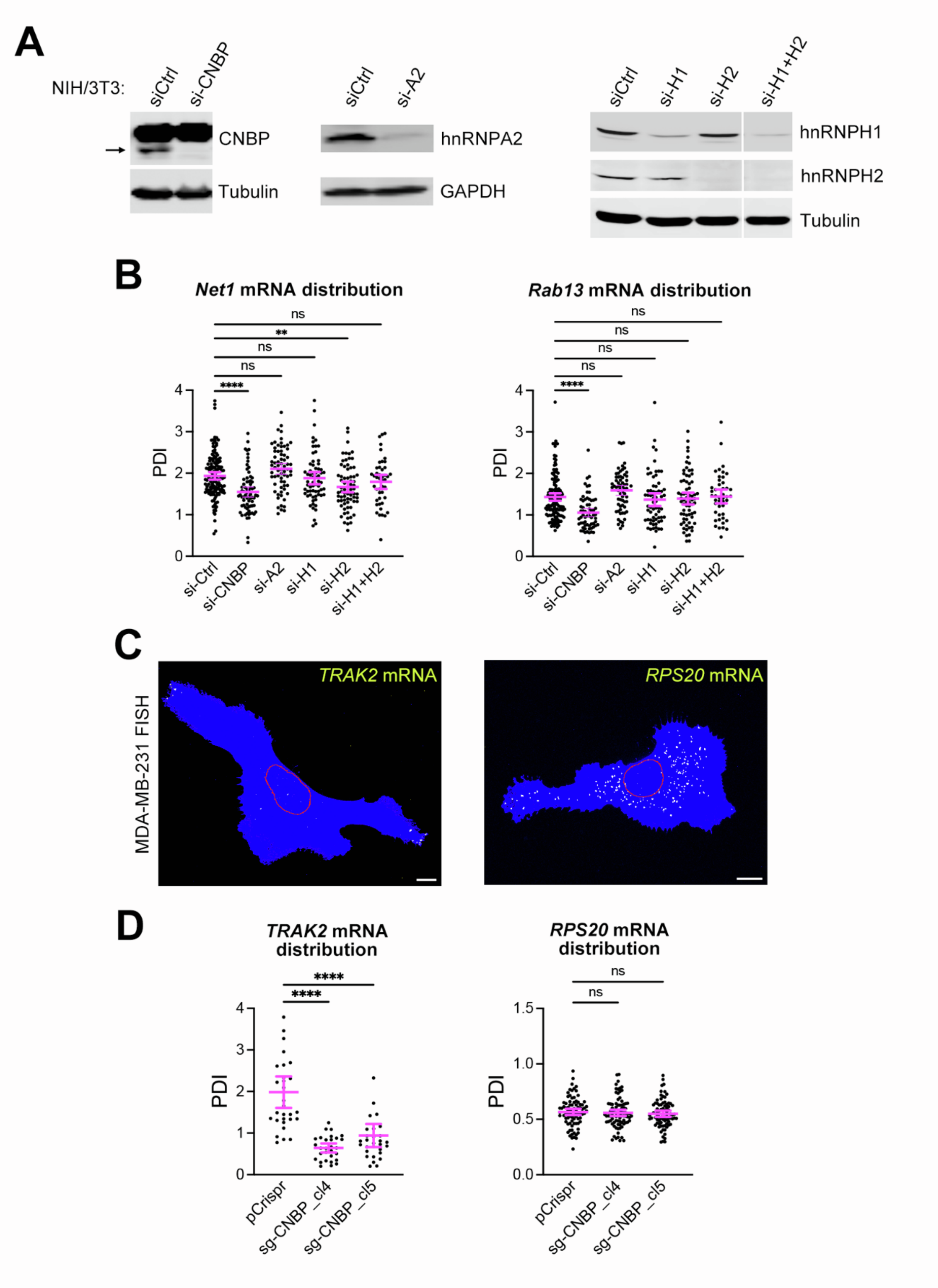

Figure S1: CNBP knockdown prevents localization of mRNAs to cytoplasmic protrusions. (Related to Figure 2). **(A)** Western blot of NIH/3T3 cells transfected with the indicated siRNAs against RBPs (CNBP, hnRNPA2, hnRNPH1 and hnRNPH2). Note that the CNBP antibody used

cross-reacts prominently with a non-specific band. Signal corresponding to CNBP is indicated by an arrow. **(B)** PDI quantifications of mouse *Rab13* and *Net1* mRNA distributions from the indicated siRNA-treated NIH/3T3 cells. n=45-136 cells. Error bars: SEM. p-values: **<0.01, ****<0.0001, ns: non-significant by Kruskal-Wallis test with Dunn's multiple comparisons test. **(C)** Representative FISH images of control (pCrispr) MDA-MB-231 cells detecting the indicated mRNAs. Blue: cell mask; Red line: outline of nucleus (based on DAPI stain); Yellow: RNA. Scale bar: 10 μm. **(D)** PDI quantifications of *TRAK2* and *RPS20* mRNA distributions from the indicated CRISPR-edited MDA-MB-231 cell lines. n=30-85 cells. Error bars: SEM. p-values: ****<0.0001, ns: non-significant, by Kruskal-Wallis test with Dunn's multiple comparisons test.

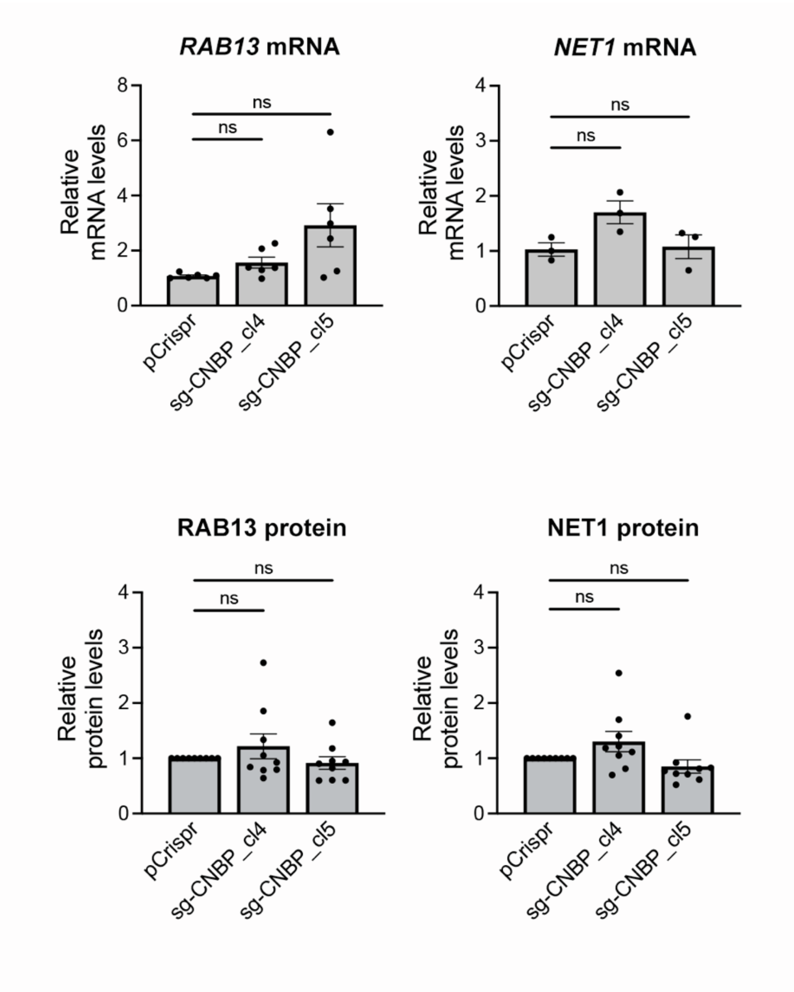

Figure S2: CNBP loss does not affect RAB13 and NET1 mRNA or protein levels. (Related to Figure 2). RAB13 and NET1 mRNA levels (measured by ddPCR) and protein levels (measured by Western blot) in the indicated CRISPR-edited clonal cell lines.

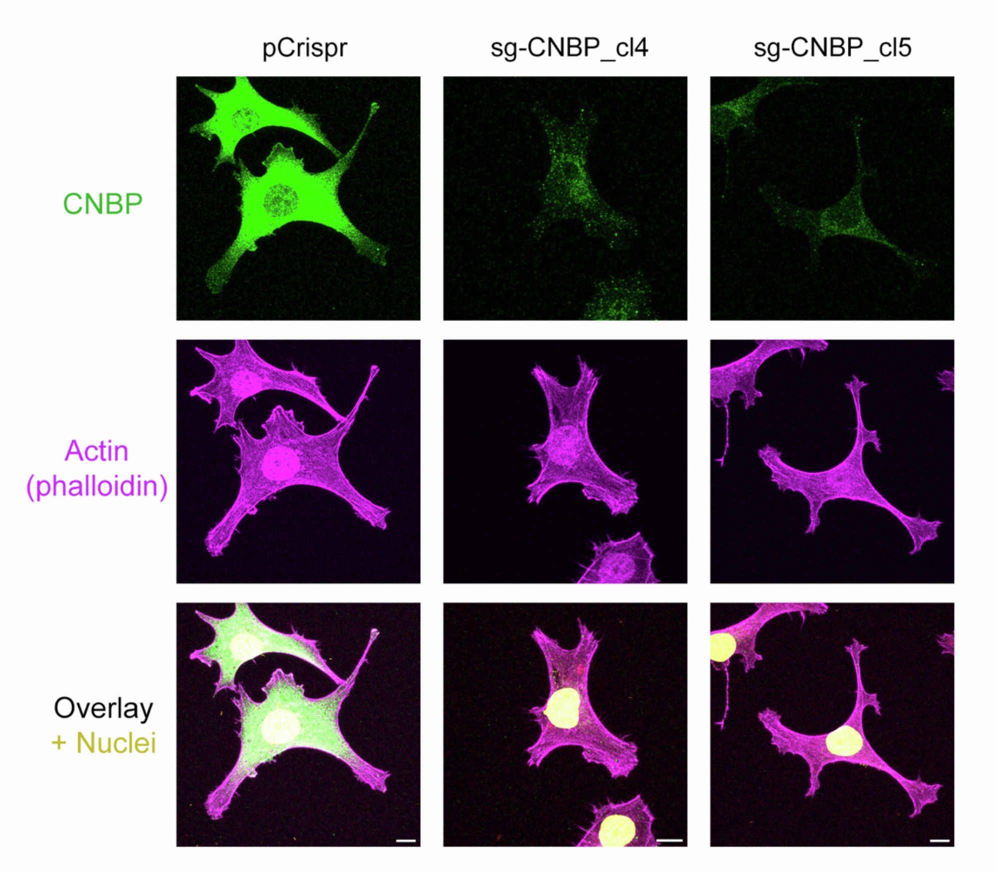

Figure S3: CNBP distributes diffusely in the perinuclear cytoplasm. (Related to Figure 3). CNBP immunofluorescence in the indicated CRISPR-edited clonal cell lines. Cells were also stained with phalloidin to visualize the actin cytoskeleton and delineate cell morphology, as well as with DAPI to visualize nuclei. Staining is specific for CNBP since it is markedly reduced in CNBP knockout clones. CNBP distributes diffusely in the cytoplasm with the bulk accumulating around the nucleus. Scale bars: 10µm.

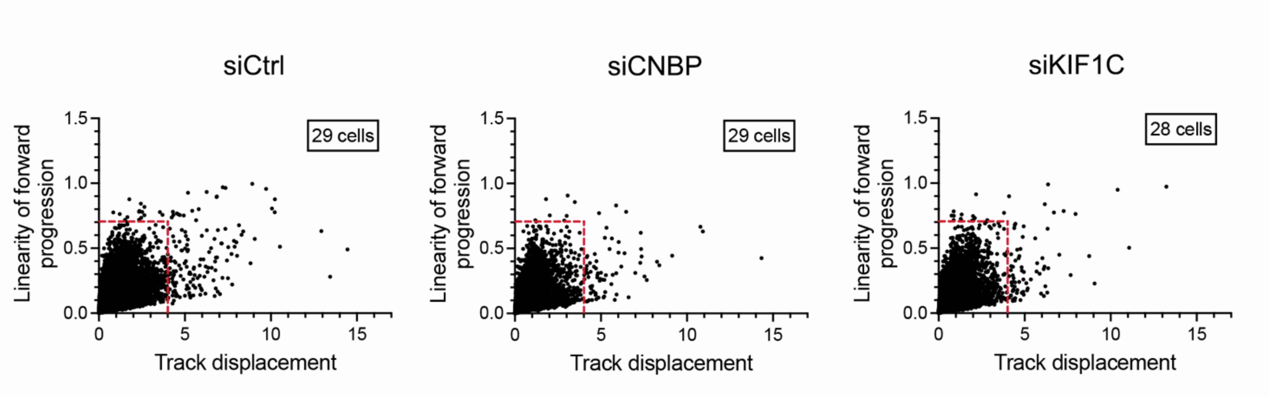

Figure S4: RNA particle tracking metrics. (Related to Figure 4). Graphs plot the displacements of all individual RNA tracks (*x*-axis) over the linearity of their forward progression (*y*-axis) (defined as the mean straight line speed divided by the mean speed), from cells expressing MS2 RNA reporter and treated with the indicated siRNAs. Representative images and particle tracking movies are shown in Fig. 4A and supplementary movies 1-3. Red lines indicate the thresholds used to filter tracks of molecules undergoing directed movement (see Methods).

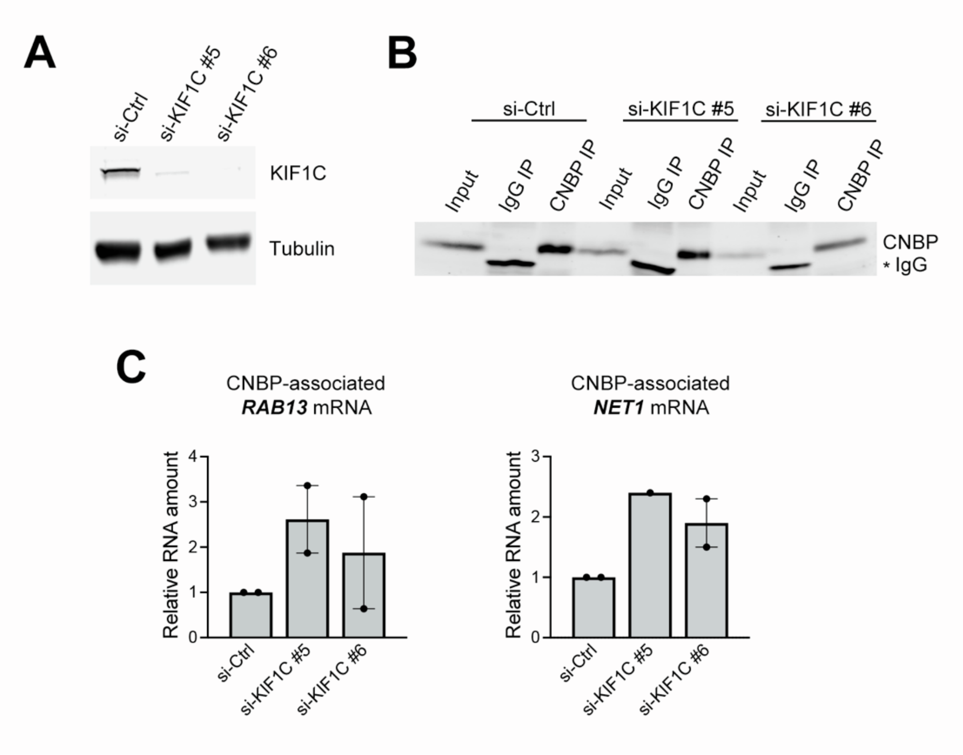

Figure S5: KIF1C is not required for binding of CNBP to protrusion-targeted mRNAs. **(**Related to Figure 5). **(A)** Western blot of MDA-MB-231 cells transfected with the indicated siRNAs. **(B)** Control or si-KIF1C transfected cells were immunoprecipitated with anti-CNBP, or control IgG, antibodies and recovered protein was analyzed by Western blot. Asterisk indicates a band originating from the IgG antibody. **(C)** Amount of *RAB13* and *NET1* mRNAs co-immunoprecipitated with CNBP from cells treated with the indicated siRNAs. Measured by ddPCR and expressed as amount of RNA in IP eluate over amount in the input normalized to control sample.

Table S2: DNA sequences used for generation of in vitro transcription templates

T7prom-MmPkp4-BoxB

AAGCTTTAATACGACTCACTATAGCATCAAGACGGCTGCCTGCTGAGGGGCGCTTT
CCTTCTGACTCTGTTTGGATTGAGGGGAAGTCCGTCTTGCTGATGATGGTGACCGT
GAAAGTGAAATGGAAGGGATGAGTGAAGAGGTTTTGGTTTGTTTGTTTGTTTTTTCT
TTTTTTGAGGAATTTTCAGGGAAGTGAGGAAACCCTTGGGAGAGGACTTTGTACGC
GCTGTGTAGGTGTTAGATCTAATTACTTGTAGAGTCTAGTGGTGAAGGTGTGGGTGA
CGTGCTGGGAGGCTTGAGACGTGGGTGAGATGAGATGGGTATGTGTAGGTCAAAT
CAAATGACAGATGATTTTTTTAATGTGAATAAAGTTATGTTCAGATAGTTTGTACAGAA
AAAATAATAATAAAATGGATGCCCTTCATGTTTTATTGCTATTACTAAATGTCAAGATTG
TATGCTATTATGTCTTGTAAAAATTCCTTCTGTTGGTGTAAATATGGAAATGCCACATT
GGTTAAGTGCCATCAATTGTAATGCAGTGTGTCATTTGAAAAGAGATTTGAAGAACT
GACAGCTTAAAGCCCAAGCGGGAAACCCGCCCGGGAACTGTTCGCAGTTGACAAC
AAACTCTGACGCCCTCTGTTTTCAGTGAGTAGTGAACTCCGGAAGCACAAAGGCCA
GCGTGACAGCAGCGCCATGCTCATCCCCCTCACAGGACACTTCACTGCCATTTTCT
ATGCACATGGAAGAATAATAAATGTGGAAATTTCATCCTGAA**GCGCCGAACT**GGGCCC
gacgactgtagaaaagggccctgaagaagggccctctgctgtctagcGAATTC

T7prom-MmPkpA-BoxB

AAGCTTTAATACGACTCACTATAGCATCAAGACGGCTGCCTGCTGAGGGGCGCTTT
CCTTCTGACTCTGTTTGGATTGAGGGGAAGTCCGTCTTGCTGATGATGGTGACCGT
GAAAGTGAAATGGAAGGGATGAGTGAAGAGGTTTTGGTTTGTTTGTTTGTTTTTTCT
TTTTTTGAGGAATTTTCAGGGAAGTGAGGAAACCCTTGGGAGAGGACTTTGTACGC
GCTGTGTAGGTGTTAGATCTAATTACTTGTAGAGTCTAGTGGTGAAGGTGTGGGTGA
CGTGCTGGGAGGCTTGAGACGTGGGTGAGATGAGATGGGAA**GCGCCGAACT**GGGC
CCgacgactgtagaaaagggccctgaagaagggccctctgctgtctagcGAATTC

T7prom-MmPkpB-BoxB

AAGCTTTAATACGACTCACTATAGCCCTTCATGTTTTATTGCTATTACTAAATGTCAA
GATTGTATGCTATTATGTCTTGTAAAAATTCCTTCTGTTGGTGTAAATATGGAAATGCC
ACATTGGTTAAGTGCCATCAATTGTAATGCAGTGTGTCATTTGAAAAGAGATTTGAAG
AACTGACAGCTTAAAGCCCAAGCGGGAAACCCGCCCGGGAACTGTTCGCAGTTGA
CAACAAACTCTGACGCCCTCTGTTTTCAGTGAGTAGTGAACTCCGGAAGCACAAAG
GCCAGCGTGACAGCAGCGCCATGCTCATCCCCCTCACAGGACACTTCACTGCCAT
TTTCTATGCACATGGAAGAATAATAAATGTGGAAATTTCATCCTGAA**GCGCCGAACT**G
GGCCCgacgactgtagaaaagggccctgaagaagggccctctgctgtctagcGAATTC

T7prom-MmRab13-BoxB

AAGCTTTAATACGACTCACTATAGAGCATTTCTTGCCTCCTATTCACCCTGAACCTG
GAGGCTAGACCTGAGGGAGTCGGACTGAGGGATTGCAGATGGGAGAACTGTGGT
GGCACCTCAAGGGGAGATGAGGGGAATAAGGAGACCGGCGAGGACGAGACGGAA
GAAAGGGGCAGGGAAAGGAGGGGGAGGAACCAAGGATGTGAAAGGTGAACAGAA
GGGATTTGAGAAGAGGAAAGGAAGAAGAAATGAATGGCTCAGGCCTTGGACAGTC
CAACATTAAAGTCAACATGCTGATCTCTCCATTCCTGGTTCAGGGTTAGGGTCCTGA
GAGGCTGGCTCGGCACTACTCCGAGGGTCCCTCACTCTACAAGGTCTTTGTTAGTA
TTAAAGGCCACTGTTTTGCATGAATGTCCCATTTGCATTACTTTCATTATTGTCAGAAT
TGCTCTTCACTCAAATCCTATTTTTGTCACGCCAAGATATTGGTTCACCTGAATGTGG
CTGGGTTCCCCTTCCTTGCCCCAACTCTTTCACTGGTGATGAAAACAGCATGGGGC
AGCCTGAAGGACGGACATCCTGTTTCCACTGTGGGTTCCCAAGGACTACAAGAGT
GGACGGAACCTTGCCTTGAGCACACAGTAACCCAAGGACAAAGGATTTGAACCAG
GCTTCAGTAAACAGCAGCACTTAGTATGGTTTATCCAAGGAGATGTGGGACATCTTT
GATTCTGATGTAGTCAGCTTAGGTGTTGGGTACTGTTAGCTGCTTTTGTTAGAGTATT
CTCAGTGTTGCACAAAGAAATACATGAACAAGGTGAA**GCGCCGAACT**GGGCCCgacga
ctgtagaaaagggccctgaagaagggccctctgctgtctagcGAATTC

T7prom-HsRAB13-BoxB

aagctt**TAATACGACTCACTATAG**GGACCCTTTCTTGCCTCCCCACCCCGGAAGCTGA
ACCTGAGGGAGACAACGGCAGAGGGAGTGAGCAGGGGAGAAATAGCAGAGGGGC
TTGGAGGGTCACATAGGTAGATGGTAAAGAGAATGAGGAGAAAAAGGAGAAAAGG
GAAAAGCAGAAAGGAAAAAAAGGAAGAGAGAGGAAGGGAGAAGGGAGAGGAATG
AATTGAGGAAGTGAAAGAAGGCAAGGAGGTAGGAAGAGAGGGAGGAGGAAAGGA
AGGAGAGATGCCTCAGGCTTCAGACCTTACCTGGGTTTTCAGGGCAAACATAAATG
TAAATACACTGATTTATTCTGTTACTAGATCAGGTTTTAGGGTCCTGCAAAAGGCTAG
CTCGGCACTACACTAGGGAATTTGCTCCTGTTCTGTCACTTGTCATGGTCTTTCTTG
GTATTAAAGGCCACCATTTGCACAAATGTTCCTGTTTTGGGTAACTTGGATTATTGTC
AGGGGCCCgacgactgtagaaaagggccctgaagaagggccctctgctgtctagcGCTAGCGCGGCCG
Cgaattc

T7prom-HsRAB13(ΔGA)-BoxB

aagctt**TAATACGACTCACTATAG**GGACCCTTTCTTGCCTCCCCACCCCGGAAGCTGA
ACCTGAGGGAGACAACGGCAGAGGGAGTGAGCAGGGGAGAAATAGCAGAGGGGC
TTGGAGGGTCACATAGGTAGATGGTAAAGAGAATGAGGAGAAAAAGGAGAAAAGG
GAAAAGCAGAAAGGAAAAAAAGGAAGAGAGAGGAAGGGAGAAGGGAGAGGAATG
AATTAGAGATGCCTCAGGCTTCAGACCTTACCTGGGTTTTCAGGGCAAACATAAATG
TAAATACACTGATTTATTCTGTTACTAGATCAGGTTTTAGGGTCCTGCAAAAGGCTAG
CTCGGCACTACACTAGGGAATTTGCTCCTGTTCTGTCACTTGTCATGGTCTTTCTTG
GTATTAAAGGCCACCATTTGCACAAATGTTCCTGTTTTGGGTAACTTGGATTATTGTC
AGGGGCCCgacgactgtagaaaagggccctgaagaagggccctctgctgtctagcGCTAGCGCGGCCG
Cgaattc
